# Structural rigidity, thermochromism and piezochromism of layered hybrid perovskites containing an interdigitated organic bilayer†

**DOI:** 10.1039/d4sc06637e

**Published:** 2025-02-24

**Authors:** Arthur Maufort, Melissa Van Landeghem, Maxime Deutsch, Peter Banks, Paola La Magna, Kristof Van Hecke, Jesús Cerdá, Laurence Lutsen, Dirk Vanderzande, Claudio Quarti, David Beljonne, Sébastien Pillet, Koen Vandewal, Wouter T. M. Van Gompel

**Affiliations:** a Hybrid Materials Design, Institute for Materials Research (imo-imomec), Hasselt University Martelarenlaan 42 B-3500 Hasselt Belgium wouter.vangompel@uhasselt.be; b Organic Opto-Electronics, Institute for Materials Research (imo-imomec), Hasselt University Martelarenlaan 42 B-3500 Hasselt Belgium; c Laboratoire de Cristallographie, Résonance Magnétique et Modélisations, Université de Lorraine, CNRS 54000 Nancy France; d Laboratory for Chemistry of Novel Materials, Materials Research Institute, University of Mons Place du Parc 20 B-7000 Mons Belgium; e XStruct, Department of Chemistry, Ghent University Krijgslaan 281-S3 B-9000 Ghent Belgium; f Imec-imomec Wetenschapspark 1 B-3590 Diepenbeek Belgium; g EnergyVille Thor Park 8310 B-3600 Genk Belgium

## Abstract

Layered hybrid perovskites are intensively researched today as highly tunable materials for efficient light harvesting and emitting devices. In classical layered hybrid perovskites, the structural rigidity mainly stems from the crystalline inorganic sublattice, whereas the organic sublattice has a minor contribution to the rigidity of the material. Here, we report two layered hybrid perovskites, (BTa)_2_PbI_4_ and (F_2_BTa)_2_PbI_4_, which possess substantially more rigid organic layers due to hydrogen bonding, π–π stacking, and dipole–dipole interactions. These layered perovskites are phase stable under elevated pressures up to 5 GPa and upon temperature lowering down to 80 K. The organic layers, composed of benzotriazole-derived ammonium cations, are among the most rigid in the field of layered hybrid perovskites. We characterize structural rigidity using *in situ* single-crystal X-ray diffraction during compression up to 5 GPa. Interestingly, the enhanced rigidity of the organic sublattice does not seem to transfer to the inorganic sublattice, leading to an uncommon material configuration with rigid organic layers and deformable inorganic layers. The deformability of the inorganic sublattice is apparent from differences in optical properties between the crystal bulk and surface. Supported by first-principles calculations, we assign these differences to energy transfer processes from the surface to the bulk. The deformability also leads to reversible piezochromism due to shifting of the photoluminescence emission peak with increasing pressure up to 5 GPa, and thermochromism due to narrowing of the photoluminescence emission linewidth with decreasing temperature down to 80 K. This raises the possibility of applying these phase-stable layered hybrid perovskite materials in temperature and/or pressure sensors.

## Abstract 2

In this manuscript, the piezochromism and thermochromism of phase-stable 2D hybrid perovskites containing a rigid organic bilayer are explored. Dual-band emission from single crystals is linked to emission from both the surface and the bulk.

## Graphical abstract

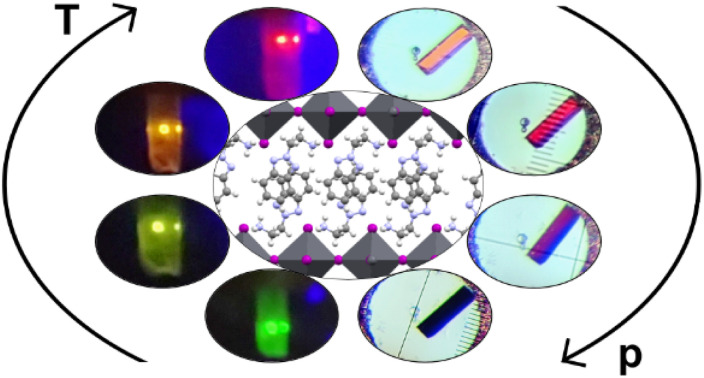

## Introduction

Layered hybrid perovskites are intensively researched today as widely tunable materials for high-performance light harvesting and emitting applications.^1–6^ They are typically self-assembled from solution at low temperatures, which is ideal for the industrial production of low-cost printable electronics.^7^ The crystal structure of these perovskites consists of atomically thin layers of corner-sharing metal halide octahedra, separated by layers of bulky organic ammonium cations. Both sublattices possess distinct frontier energy levels and dielectric constants, causing quantum and dielectric confinement.^8–11^ As a result, the excited states of layered hybrid perovskites can best be described by excitons, *i.e.* bound electron–hole pairs generally localized in the inorganic sublattice.^12,13^ The spatial and dielectric confinement cause the exciton binding energy in layered hybrid perovskites to reach hundreds of meV, significantly larger than in their three-dimensional counterparts.^8,9^

The organic sublattice plays an important role in layered hybrid perovskites. Firstly, it affects the metal–halide bond lengths and angles and the degree of octahedral tilting, which dictates the band gap and electronic band structure of the perovskite.^12,14–16^ Secondly, several reports have demonstrated the favorable influence of carefully designed organic layers on various properties, including charge carrier mobility,^17–20^ charge and energy transfer,^21–28^ exciton binding energy,^29^ and environmental stability.^30–32^ Layered hybrid perovskites excel in the tunability of the organic moiety: the organic ammonium cations can be selected from a nearly endless library.^33–35^

Additionally, the organic cations impact the rigidity of the perovskite structure. Engineering the intermolecular cohesive forces in the organic sublattice opens the door towards substantially more rigid organic layers and, hence, to more rigid layered hybrid perovskites. For example, Gong *et al.* found that phenethylammonium (PEA) derivatives form more rigid lead(ii) bromide perovskites than alkylammonium derivatives due to CH–π interactions.^36^ Seitz *et al.* recently observed the same for lead(ii) iodide perovskites.^37^ Additionally, Denis *et al.* designed organic layers based on benzothieno[3,2-*b*]benzothiophene (BTBT) chromophores, possessing much stronger intermolecular interactions than PEA cations, which greatly improved the thermal stability of the perovskite structure.^30^ Other studies have further demonstrated this relationship between rigidity and thermal stability.^38,39^ Conceptually, weakly interacting organic compounds, such as PEA and alkylammonium derivatives, form soft perovskites that show disorder in the organic sublattice and/or phase transitions at comparatively low pressures or temperatures.^40–42^ In contrast, more tightly interacting organic cations are expected to crystallize into stiffer perovskite structures. We anticipate that strong intermolecular interactions in the organic sublattice facilitate the self-assembly of the perovskite structure and increase the phase stability. Ultimately, this would lead to phase-stable layered hybrid perovskites with tunable optical properties within a wide temperature–pressure range.

In previous studies,^43,44^ we have shown that benzotriazole-based lead(ii) iodide perovskites possess a considerable extent of cohesive interactions in the organic sublattice, namely inter- and intramolecular hydrogen bonding, dipole–dipole interactions, and π–π stacking. Additionally, these perovskites show interdigitation in the organic sublattice, leading to the absence of the van der Waals gap that is typically present in layered hybrid perovskites with monoammonium cations. Here, we assess the impact of these strong organic intermolecular interactions on the rigidity of layered hybrid perovskites. We do so with a diverse set of complementary characterization techniques: we follow the structural evolution of layered hybrid perovskites under pressure using *in situ* single-crystal XRD; furthermore, we combine single-crystal photoluminescence (PL) emission and X-ray diffraction (XRD) data in a wide temperature and pressure range; additionally, we support our observations with density-functional theory (DFT) modelling, unraveling the relationship between the crystal structure and the optical properties. We determine that the multitude of organic interactions leads to a rigid organic sublattice. At the same time, however, this enhanced rigidity does not transfer to the inorganic sublattice. This lack of rigidity transfer likely stems from a structural decoupling of the organic and inorganic layers, as the ammonium group-mediated interactions between the two sublattices are weaker than the intramolecular hydrogen bonding within the organic sublattice.^44^ Interestingly, this decoupling results in atypical structural properties. The deformable inorganic sublattice shows marked piezochromism and thermochromism, causing the crystal color to change from orange to red to brown with increasing pressure, and from orange to yellow to green with decreasing temperature. At the same time, these materials are phase-stable within the entire pressure–temperature domain under study, up to 5 GPa and down to 80 K, resulting in a continuous modulation of the optical properties as a function of pressure and temperature. The combination of phase stability with an easily deformable inorganic sublattice provides prospects for the application of these hybrids in temperature and/or pressure sensors. Additionally, this work demonstrates the synergy that can be achieved between the organic and inorganic moieties of layered hybrid perovskites through careful molecular engineering.

## Results and discussion

### Crystallography under elevated pressure

**Fig. 1:**
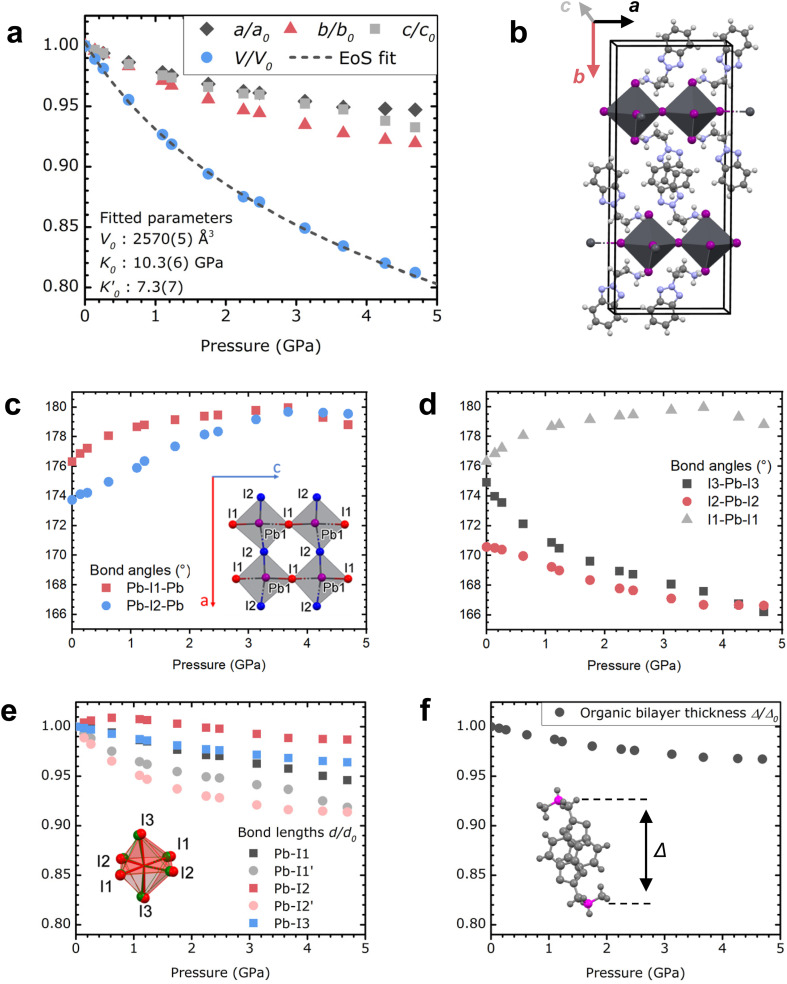
Evolution of the crystal structure of (BTa)_2_PbI_4_ under pressure in the 0–5 GPa domain. (a) Lattice parameters and the unit cell volume, which are defined in (b). The decrease in unit cell volume is fitted with a third-order Birch–Murnaghan equation of state (EoS). Pressure points above the pressure oil's hydrostatic limit (at 3.7 GPa) were not used for the EoS fit. (c) Pb–I–Pb bond angles, (d) I–Pb–I bond angles, (e) Pb–I bond lengths, and (f) thickness of the organic bilayers as a function of pressure, as indicated in the inset figures.

We recently reported benzotriazole-based layered hybrid perovskites, which show great cohesive interaction in the organic sublattice. Hereafter, we find that such organic cohesion reduces the amount of disorder and eliminates phase transitions in the 0–5 GPa pressure range, in contrast to alkylammonium and PEA perovskites.^40,42^ Single crystals of two perovskite derivatives have been studied here: (BTa)_2_PbI_4_ and (F_2_BTa)_2_PbI_4_, with BTa = 2-(2*H*-benzo[*d*][1,2,3]triazol-2-yl)ethylammonium and F_2_BTa = 2-(5,6-difluoro-2*H*-benzo[*d*][1,2,3]triazol-2-yl)ethylammonium. While previous studies performed powder XRD measurements of layered hybrid perovskites under pressure,^45–48^ the high quality of the single crystals enabled us to use single-crystal XRD to follow their structural evolution *in situ* while varying the external pressure between 0 and 5 GPa; the results are shown in Fig. 1 for (BTa)_2_PbI_4_ and in Fig. S1† for (F_2_BTa)_2_PbI_4_. We attribute the absence of phase transitions in the measurement range to the increased organic cohesive interaction. Furthermore, the structural changes that occur as the pressure increases to 5 GPa are fully reversed after the pressure is released. In fact, the data points at 0.14 GPa, 1.23 GPa, and 2.48 GPa in Fig. 1 (0.10 GPa, 1.61 GPa, and 3.90 GPa in Fig. S1†) were measured while releasing the pressure and are perfectly in line with the observed trends.

The evolution of the unit cell parameters and volume with pressure is plotted in Fig. 1a; the unit cell parameters of (BTa)_2_PbI_4_ are defined in Fig. 1b. We observe a volume contraction of 18% between 0 and 4.69 GPa. Below the hydrostatic limit of the pressure transmitting medium, at 3.6 GPa, the contraction of the *a* and *c* parameters is equivalent (*a* 5.1%, *c* 5.3%) and substantially lower than the contraction of the *b* parameter (7.2%, Fig. S2†), the latter coinciding with the layer stacking direction. The volume contraction was fitted with a third-order Birch–Murnaghan equation of state (EoS), yielding a reference volume *V*_0_ = 2570(5) Å^3^, a bulk modulus *K*_0_ = 10.3(6) GPa, and a first derivative of the bulk modulus 
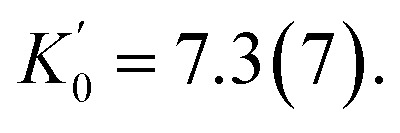
 (F_2_BTa)_2_PbI_4_ shows a similar contraction under pressure (Fig. S1†) with a slightly increased reference volume and bulk modulus: *V*_0_ = 2613(5) Å^3^ and *K*_0_ = 12.3(6) GPa. This increase complies with the anticipated effect of the fluorine substituents in (F_2_BTa)_2_PbI_4_, which enlarge the unit cell volume and give rise to additional cohesive energy and related structural stiffening through intermolecular hydrogen bonding in the organic layers, which is not present in (BTa)_2_PbI_4_.^44^ A bulk modulus of 10–12 GPa is in the range of typical 2D perovskites (∼7–28 GPa).^49^ For example, the first phase of benzylammonium lead iodide, which exists from ambient pressure up to 0.78 GPa, possesses a bulk modulus of 11.4 GPa (as derived from a second-order Birch–Murnaghan EoS).^49^ This shows that, overall, the benzotriazole perovskites are as compressible as typical 2D perovskites. However, the behavior of the organic sublattice of the benzotriazole perovskites is peculiar (*vide infra*).

We now focus on the local changes in the organic and inorganic sublattices as the pressure increases. The structural evolution with pressure is illustrated in Fig. 1c–f. Briefly, the organic layers remain relatively unaffected under pressure, whereas significant changes occur in the inorganic part of these hybrids. With increasing pressure, the organic cations are pushed deeper into the inorganic layers (Fig. S3 and supporting movies†), modifying the inorganic layers and deforming the Pb–I octahedral shape. As shown in Fig. 1c, the angles by which the octahedra are connected (Pb–I–Pb) approach 180° under increasing pressure up to 3.67 GPa. However, the main change is observed in the angles within the octahedra (Fig. 1d and S4†) and, more specifically, I3–Pb–I3 in the apical direction, which decreases from 174.9° to 166.2° between 0 and 4.69 GPa. This leads to an increase in the bond angle variance (Fig. S5†). Furthermore, the Pb–I bonds shrink with increasing pressure, decreasing the bond length distortion index (Baur elongation parameter,^50^Fig. 1e and S5†).

Now we turn our focus to the organic sublattice. Fig. 1f depicts the contraction of the organic sublattice, represented by the distance *Δ* between the carbon atoms that are bound to their respective ammonium groups in a face-stacked benzotriazole couple. We opted for these carbon atoms instead of the ammonium groups themselves in order to include the full width of the organic layer, since the ammonium groups turn back out of the inorganic sublattice to form intramolecular hydrogen bonds with the benzotriazole core.^44^ In Fig. 1f, *Δ* is only reduced by 3% of its initial value as the pressure increases from 0 to 4.69 GPa. The contrast with the distortion of the inorganic sublattice (7.2%) is remarkable, especially since Fig. 1c–e depict individual ionic inorganic bonds and angles, whereas Fig. 1f depicts an ensemble of covalent bonds and non-covalent interactions. Similar observations are made for (F_2_BTa)_2_PbI_4_ (Fig. S1†), leading to the same conclusion that the organic sublattice appears substantially more pressure-resistant than the inorganic sublattice.

**Fig. 2:**
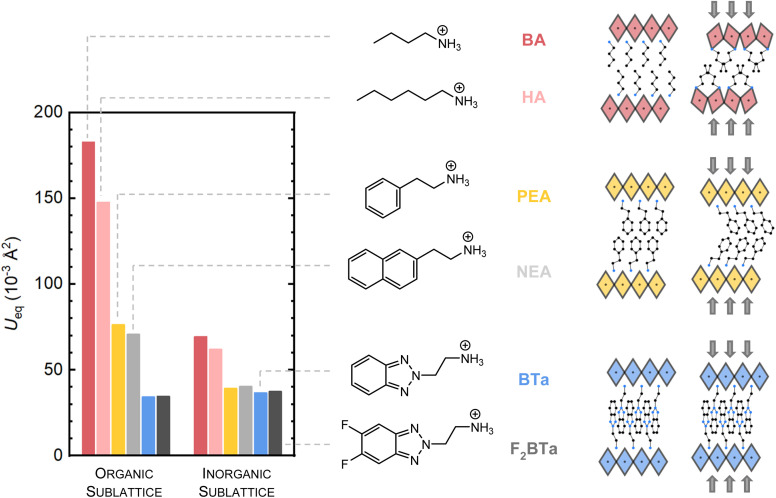
Average isotropic atomic displacement (*U*_eq_) in the organic and inorganic sublattices of six lead(ii) iodide layered hybrid perovskites at room temperature. The difference in atomic displacement can be related to the different behavior of the crystal structure under elevated pressure. Moving from (BA)_2_PbI_4_ to (PEA)_2_PbI_4_ and then to (BTa)_2_PbI_4_, the *U*_eq_ values drop, and the relevant sublattices become increasingly more pressure-resistant.

To support these findings, we investigated the equivalent isotropic displacement (*U*_eq_) parameters of the atoms in the crystal structure at ambient pressure and temperature, determined *via* single-crystal XRD. Such parameters indicate the extent of dynamic atomic displacement within the crystal structure. It has been shown in the literature that rigid perovskite structures typically possess low *U*_eq_ parameters.^36,37^Fig. 2 compares the average *U*_eq_ values of (BTa)_2_PbI_4_ and (F_2_BTa)_2_PbI_4_ with four literature references, namely (BA)_2_PbI_4_,^51^ (HA)_2_PbI_4_,^51^ (PEA)_2_PbI_4_,^14,52^ and (NEA)_2_PbI_4_,^14^ all of which show less cohesive interaction in the organic layers. The crystal structures of these six perovskites are depicted in Fig. S6;† a detailed overview of the *U*_eq_ values per element for all six compounds is given in Table S4.†

In Fig. 2, the rigidity increase in the organic sublattice complies with the enhanced organic intermolecular interactions. (PEA)_2_PbI_4_ and (NEA)_2_PbI_4_ possess a more rigid organic sublattice than the alkylammonium perovskites due to CH–π interactions and a more space-filling packing, as established by Seitz *et al.*^37^ (NEA)_2_PbI_4_ only has a slightly more rigid organic sublattice than (PEA)_2_PbI_4_, although its aromatic system is larger, indicating that these organic layers do not pack appropriately to allow for strong π–π interactions, as is also evident from the crystal structure. The BTa- and F_2_BTa-based organic layers, in contrast, show more efficient π–π stacking (with a previously calculated interaction energy of 6.0 kcal mol^−1^)^44^ than PEA and NEA because of interdigitation in the organic layers (Fig. S6†), which is reflected in the 50% lower *U*_eq_ value of the organic layers. Moreover, these benzotriazole perovskites show intramolecular hydrogen bonding (4.0 kcal mol^−1^, Fig. S6†),^44^ which likely adds to the rigidity improvement. Although (F_2_BTa)_2_PbI_4_ additionally contains intermolecular hydrogen bonding (1.4 kcal mol^−1^),^44^ both benzotriazole perovskites appear equally rigid at ambient temperature based on *U*_eq_ values; at 100 K, however, we measured a 30% increase in rigidity of the organic sublattice of (F_2_BTa)_2_PbI_4_ over (BTa)_2_PbI_4_ (Table S5†). From the perspective of *U*_eq_ values and compressibility, the BTa-based and F_2_BTa-based organic layers are among the most rigid in the field of layered hybrid perovskites.

We now move to the rigidity of the inorganic sublattice. This time, interestingly, the *U*_eq_ parameters of the benzotriazole perovskites are at the same level as those of (PEA)_2_PbI_4_ and (NEA)_2_PbI_4_ (Fig. 2). Typically, an increase in the rigidity of the organic layer translates into an increased rigidity of the inorganic sublattice.^36,53^ However, for the benzotriazole perovskites, this is not the case. In fact, at room temperature, the values are similar, and at 100 K both benzotriazole perovskites have a higher average *U*_eq_ than (PEA)_2_PbI_4_ for the inorganic sublattice (Table S5†). We hypothesize that this is related to weakened hydrogen bonding between the ammonium groups and the inorganic sublattice in the benzotriazole perovskites, which is apparent from their crystal structure (Fig. S6†). The lack of strong interaction between the organic and inorganic moieties might cause the transfer of rigidity to be hampered. One should, however, be careful to use *U*_eq_ parameters for analyzing the rigidity of the structure, since *U*_eq_ parameters account at the same time for a dynamic atomic displacement and a static disorder intrinsic to the samples. Nevertheless, our findings seem to support the high-pressure single-crystal XRD results discussed above. The benzotriazole perovskites behave markedly different under pressure compared to (BA)_2_PbI_4_ and (PEA)_2_PbI_4_, which have been thoroughly investigated in the past.^45,46^ The key differences are illustrated schematically on the right-hand side of Fig. 2. Alkylammonium perovskites readily undergo phase transitions under pressure due to a lack of strong organic intermolecular interactions and a low overall structural rigidity. In the prototypical butylammonium lead iodide 2D perovskite,^54^ significant changes in the conformation of the organic cations occur under pressure in concordance with phase transitions at 0.22 GPa and 2.2 GPa. In the benzotriazole perovskites, no phase transition occurs up to at least 5 GPa. In (PEA)_2_PbI_4_, Liu *et al.* found that the organic moiety is easily deformable and that the PEA cations act like springs, while the inorganic moiety barely shows any deformation.^45^ Similarly, for benzylammonium lead bromide, Feng *et al.* showed using experiment-aided DFT calculations that the benzylammonium cations undergo a significant change in conformation between ambient pressure and 3.79 GPa, with the benzene ring tilting towards the inorganic plane.^55^ Unlike typical 2D perovskites containing a van der Waals gap in the organic layer, the benzotriazole perovskites possess an interdigitated organic layer that is hardly compressed. At the same time, the transfer of rigidity to the inorganic sublattice is hampered. The ammonium groups are pushed into the Pb–I octahedral layers, thereby changing the octahedral shape (Fig. S3†). Therefore, the inorganic layers seem to be more receptive to external pressure changes than classical layered hybrid perovskites. However, it is difficult to trace the exact differences compared to, for example, (PEA)_2_PbI_4_, for which conclusions have been drawn from a DFT model based on changes in powder XRD patterns rather than directly from *in situ* single-crystal XRD.^45^ More studies using *in situ* single-crystal XRD under pressure are required to make detailed comparisons.

### Single-crystal PL spectroscopy and exciton–phonon coupling

**Fig. 3:**
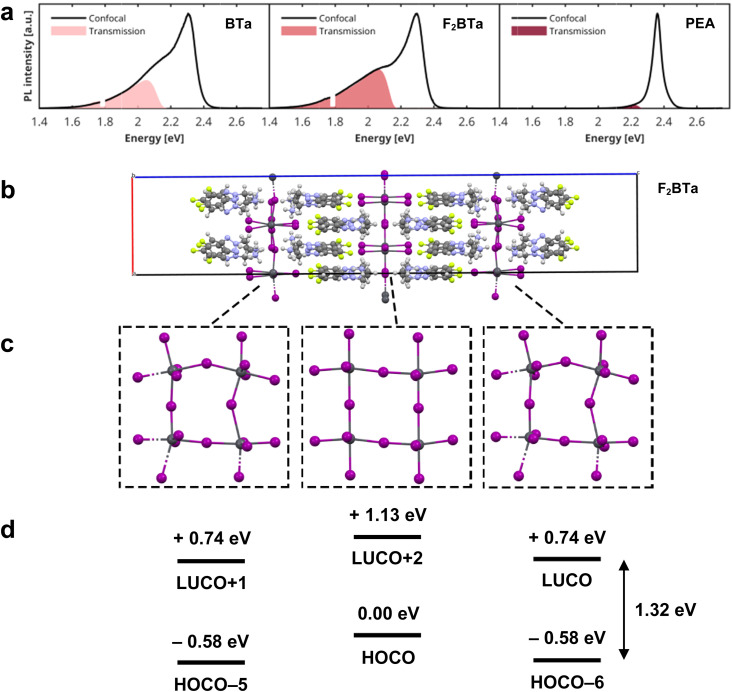
(a) Single-crystal PL spectra of (BTa)_2_PbI_4_, (F_2_BTa)_2_PbI_4_, and (PEA)_2_PbI_4_ at room temperature after excitation at 3.06 eV (405 nm), comparing the PL spectra collected using two different measurement geometries: confocal (solid line) and transmission geometry (shaded area); see the main text for details. At 1.79 eV, a narrow artifact due to a luminescent impurity in the cryostat window has been removed from all spectra. (b) Optimized structure of the (F_2_BTa)_2_PbI_4_ theoretical surface model. (c) Enlarged images of the inorganic framework at surface and bulk positions to illustrate structural distortion. (d) Diagram of the lowest-energy HOCO/LUCO transitions involving only the surface and bulk inorganic groups, respectively.

It has been shown that rigidity affects the PL behavior and exciton–phonon coupling of 2D layered perovskites: rigid perovskite structures generally show weaker coupling of excitons to phonons since the perovskite structure is less deformable.^36^ Based on the reduced equivalent isotropic displacement (*U*_eq_) parameters for the benzotriazole perovskites, especially in the organic layer, we hypothesized that these perovskites may possess reduced exciton–phonon coupling as compared to (PEA)_2_PbI_4_. Before the analysis of exciton–phonon coupling, we first examine the PL emission spectra. Fig. 3a shows representative PL emission spectra of BTa-, F_2_BTa-, and PEA-linked perovskites at room temperature. All three PL spectra are dominated by emission centered at 2.30–2.36 eV (525–540 nm), ascribed to the radiative decay of excitons within the Pb–I octahedral layers. However, unlike (PEA)_2_PbI_4_, the BTa- and F_2_BTa-linked perovskites exhibit a highly asymmetric emission line shape towards lower energies, suggestive of multiple overlapping transitions contributing to the total emission profile. This is reminiscent of the so-called dual-band emission that has been reported for other layered perovskites, particularly for BA-linked systems.^56–59^ Although the origin of this multi-component emission has been hotly debated in the literature, most recent studies agree that the two peaks arise from a difference in band gap between the (sub)surface layers and the bulk of these single crystals, due to small structural changes in the local crystalline environment.^56,58,59^ This seems to be the case for (BTa)_2_PbI_4_ and (F_2_BTa)_2_PbI_4_ as well, based on the following experimental observations: (i) we observe no saturation of the PL intensity as a function of the laser fluence, ruling out emission due to defects or trap states (Fig. S7†). (ii) The time-decay of both the high-energy and low-energy parts of the PL spectra has similar time constants, as observed in the time-resolved PL (TRPL) measurements shown in Fig. S8.† Exponential fitting of the PL decay yields a time constant of 24 ns for the high-energy peak around 2.25 eV and 29 ns for the low-energy broad emission band. Both are within the expected time range for excitonic transitions, again ruling out any longer-lived defect-related origin of the emission. (iii) Fig. 3a compares confocal PL spectra, with excitation and PL collection from the same side of the crystal, to transmitted PL spectra, in which the crystal is excited from the back. If the high energy emission peak originates from the surface of the crystal, it is expected to be reduced in the spectrum measured on the backside of the crystal. A detailed rationale behind this statement is provided in Fig. S9.† Indeed, as shown in Fig. 3a, the transmitted PL spectra only contain the low-energy part of the confocal PL spectrum, clearly indicating that the high-energy emission originates from the surface layers of the single crystal, whereas the low-energy tail arises from the bulk. Since in the transmission geometry all collected emission has traveled throughout the entire crystal, the complete absence of any high-energy surface emission suggests its elimination from the spectrum due to either bulk reabsorption^60–62^ or Förster resonant energy transfer (FRET) of the high-energy excitons towards the interior of the crystal.^63^

The observed variation in bandgap between the surface and bulk of the crystals is, of course, directly related to the inherently soft nature of hybrid organic–inorganic perovskites. Earlier computational studies have shown that even minor changes in the Pb–I–Pb bond angle, caused by, for example, microstrain due to the packing of the organic linkers, can have a significant impact on the band gap.^64,65^ To provide computational feedback on the band gap difference between the crystal bulk and surface, we created a slab model of the (F_2_BTa)_2_PbI_4_ perovskite in Fig. 3b and performed periodic DFT calculations. This model is composed of three inorganic layers terminated by F_2_BTa organic spacer molecules, including a vacuum spacing between the periodic replicas along the direction normal to the inorganic planes (Fig. 3b). This design imposes unique geometries of the ammonium and inorganic moieties that may occur at the crystal surface, which are not observed in the bulk material.

Following optimization of the atomic positions, strong distortion is observed in the surface inorganic layers, while the inner bulk framework retains a more rigid linear orientation (Fig. 3c). Specifically, the surface inorganic components relaxed to geometries yielding a maximum Pb–I–Pb angle of 163.7°, while the bulk framework yields a maximum value of 178.0°. Consequently, the highest occupied-lowest unoccupied crystal orbital (HOCO–LUCO) transition in a bulk inorganic layer corresponds to an energy gap of 1.13 eV (at the PBE level including spin–orbit coupling), while the HOCO–LUCO transition in the surface inorganic layers is predicted with a gap of 1.32 eV, which is illustrated schematically in Fig. 3d. The difference in band gap between the surface and bulk layers can be correlated to the degree of structural distortion in these regions, which has been demonstrated to result in larger band gaps for these systems.^66^ Our analysis is in line with the findings of Sheikh *et al.*,^67^ who analyzed dual peak emission in butylammonium lead iodide, (BA)_2_PbI_4_, crystals. They obtained two emission peaks for bulk (BA)_2_PbI_4_ crystals at 2.20 eV and 2.38 eV, which are close to 2.13 eV and 2.30 eV we obtained for (BTa)_2_PbI_4_. It is likely that the exact peak position of the two peaks is strongly dependent on the degree of distortion of the inorganic framework in both the bulk and at the surface, since the excitonic peak position is known to depend on the Pb–I–Pb angle.^14^ The relative intensity of the two emission peaks is expected to be dependent on the thickness of the crystal sample, which determines the ratio between the surface and bulk. Indeed, Sheikh *et al.* showed that bulk crystals of (BA)_2_PbI_4_ possess dual peak emission, while exfoliated flakes possess only a single excitonic emission peak corresponding to the ‘surface’ contribution for the bulk crystals.^67^ Moreover, the emission spectrum for a thicker bulk crystal of (PEA)_2_PbI_4_ as obtained by Jin *et al.*^63^ shows a clearer contribution from the second emission peak as compared to our sample, for which the relative intensity of the second peak is small (Fig. S11†). It can be hypothesized that the relative intensity of the surface and bulk emission peaks, for crystal samples of equal thickness, is also dependent on the extent to which the Pb–I–Pb angles differ between the surface and bulk (Fig. 3), which may, in turn, depend on the specific crystal structure of a 2D perovskite, but we are not able to draw a solid conclusion on this aspect based on our current experiments.

**Fig. 4:**
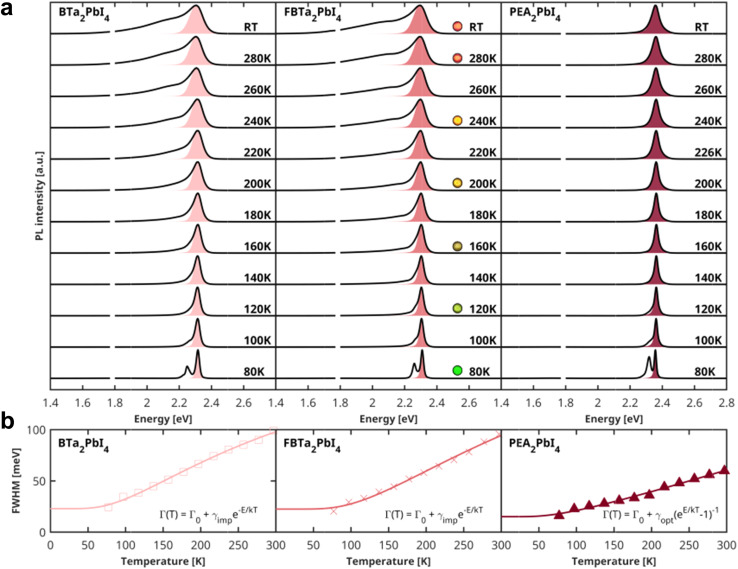
(a) Temperature-dependent PL spectra of (BTa)_2_PbI_4_, (F_2_BTa)_2_PbI_4_, and (PEA)_2_PbI_4_ single crystals (solid line) and the corresponding Voigtian fit of the high-energy flank of the emission (shaded area). The best-fit parameters for the Gaussian and Lorentzian line broadening parameters are listed in Table S6.† For (F_2_BTa)_2_PbI_4_, photographs of the emission color are added for selected temperatures. At 1.79 eV, a narrow artifact due to a luminescent impurity in the cryostat window has been removed from all spectra. (b) Temperature dependence of the FWHMs extracted from the Voigtian fits. The solid lines are fits of the line broadening with eqn (1). The best-fit parameters are summarized in Table 1.

To further investigate the rigidity of the perovskite single crystals under study, we performed temperature-dependent PL measurements in a temperature range of 80–300 K, as shown in Fig. 4a. The temperature-dependent broadening of the excitonic PL linewidth is commonly used to quantify the exciton–phonon coupling in several 3D and layered lead halide perovskites,^68,69^ which can be correlated with the rigidity of the inorganic sublattice. The total PL linewidth in these materials is composed of both homogeneous and inhomogeneous broadening terms and can be described using the equation:^68^1
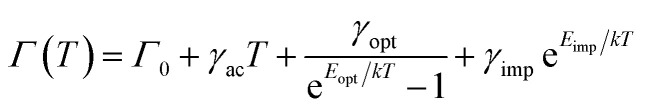
Here, *Γ*_0_ represents the temperature-independent inhomogeneous broadening due to static disorder. In the second and third terms, *γ*_ac_ and *γ*_opt_ denote the scattering strength from acoustic and optical phonons, respectively, and *E*_opt_ is the mean energy of the optical phonons. The last term phenomenologically accounts for scattering from ionized impurities,^69^ characterized by coupling constant *γ*_imp_ and ionization energy *E*_imp_.

In order to determine the dominant broadening mechanism, it is important to consider the shape of the emission profile. Homogeneous broadening mechanisms such as phonon scattering give rise to a Lorentzian line shape, whereas inhomogeneous broadening, which can be caused by disorder or scattering from ionized impurities, leads to a Gaussian emission profile. The overall line shape is, therefore, best described using a Voigtian function, which results from the convolution of the homogeneous and inhomogeneous broadening contributions to the emission peak. At the low-energy side of the PL spectra, the line shape is distorted due to the abovementioned photon recycling and/or FRET, which is why we have determined the linewidth of the spectra by fitting only the high-energy flank of each emission spectrum with a Voigtian function (Fig. 4a). We extracted the full width at half maximum (FWHM) from the best fit (see Table S6† for fit parameters); the extracted FWHM as a function of temperature is displayed in Fig. 4b. For (PEA)_2_PbI_4_, the emission peak has a clear Lorentzian profile, indicating that phonon scattering is the dominant broadening mechanism. Moreover, as shown in Fig. 4b, an excellent fit of the temperature-dependence of the FWHM for (PEA)_2_PbI_4_ can be obtained by taking only the scattering from optical phonons into account, since the contribution from acoustic phonons is typically negligible. The best-fit parameters are summarized in Table 1. The exciton–phonon coupling constant of 51 meV and optical phonon energy of 19 meV for PEA_2_PbI_4_ are in good agreement with earlier studies.^70–72^ In contrast to (PEA)_2_PbI_4_, the room-temperature PL spectra of the (F_2_)BTa-linked perovskites exhibit a Gaussian profile (Table S6† contains the fit parameters). Due to this dominant Gaussian profile, the contribution of optical-phonon scattering to the broadening is difficult to assess for (F_2_)BTa-linked perovskites (Fig. S10†). The contribution is potentially minimal since the temperature-dependent broadening can be well reproduced by only taking the term that can be phenomenologically assigned to impurity scattering into account, as shown in Fig. 4b. The obtained ionization energies (*E*_imp_) are 27 meV and 34 meV for (BTa)_2_PbI_4_ and (F_2_BTa)_2_PbI_4_, respectively, matching with active ionizable impurities that would be near-isoenergetic with the band extrema. Earlier computational studies on intrinsic defects in butylammonium-linked perovskites have identified halide vacancies as donor-type defects, which introduce a very shallow trap state near the conduction band minimum.^73^ Hence, combined with their low formation energy, iodide vacancies might be responsible for the observed broadening due to impurity scattering in (BTa)_2_PbI_4_ and (F_2_BTa)_2_PbI_4_. Nonetheless, the PL linewidth broadening being dominated by impurity scattering would be highly unusual for metal halide perovskites, in which optical-phonon scattering is usually dominant to the extent that the contribution by scattering from ionized impurities can be ignored.^41,69,74^ There can, however, be another explanation for the Gaussian profile of the emission for the benzotriazole perovskites. A condensed phase system can be described by a continuous distribution of oscillators characterized by two parameters that quantify the nuclear dynamics and the magnitude of the fluctuations. As the dimensionless ratio between these parameters is varied, the optical absorption or fluorescence spectra change continuously from a Gaussian profile in the static limit to a Lorentzian profile in the fast modulation limit.^75^ Thus, while in the lead halide perovskite community, Gaussian lineshapes are commonly associated with impurities, this is not necessarily the case. We would also expect Gaussian lineshapes in the case of slow modulation of the 2D inorganic lattice excited-state electronic structure due to a quasi-static distribution of locked molecular conformations of the organic spacers.

**Table 1:** Best-fit parameters extracted from the analysis of the temperature-dependence of the FWHMs of the emission spectra in Fig. 4a with eqn (1)

	*Γ*_0_ [meV]	*γ*_opt_ [meV]	*E*_opt_ [meV]	*γ*_imp_ [meV]	*E*_imp_ [meV]	*R* ^2^
(BTa)_2_PbI_4_	22.9	—	—	210.9	26.7	1.00
(F_2_BTa)_2_PbI_4_	22.5	—	—	269.2	34.1	0.99
(PEA)_2_PbI_4_	15.1	50.8	19.4	—	—	0.99

We have established from XRD in Fig. 2 that the inorganic layers of (BTa)_2_PbI_4_, (F_2_BTa)_2_PbI_4_, and (PEA)_2_PbI_4_ have similar structural rigidity. In contrast, the PL study in Fig. 4 and Table 1 indicates a significant difference in both the overall magnitude and the dominant scattering mechanism of the temperature-dependent emission line broadening. This discrepancy might tentatively be explained by different organic sublattice phonon modes being active in (F_2_)BTa-linked *versus* PEA-linked perovskites. Several literature studies have reported that phonon modes in the organic sublattice couple to excitons in the inorganic sublattice.^57,76–79^ Indeed, an explicit link has been made in the literature between exciton–phonon coupling in 2D hybrid organic–inorganic perovskites and the vibrational modes of C–N and NH_3_^+^ bonds of the ammonium headgroup that is typically bound to the inorganic framework *via* hydrogen bonds.^57,78^ Due to the formation of an intramolecular hydrogen bond between the ammonium headgroup and a nitrogen atom of the benzotriazole core, the typical hydrogen bond pattern between the ammonium group and the inorganic framework is absent for the benzotriazole perovskites. Furthermore, the uncommon interdigitated organic bilayer could result in the presence of locked molecular conformations, inducing a Gaussian lineshape. While such aspects are hypothesized to influence the PL lineshape in these 2D perovskites, a detailed analysis of phonon modes in benzotriazole perovskites is beyond the scope of the current study and can be the subject of future work.

Additionally, the dual-band structure of the PL spectra is much better resolved at 80 K, as can be seen in Fig. 4a. This is also the case for our (PEA)_2_PbI_4_ sample, although it did not show any significant asymmetric line broadening towards lower energies at room temperature. As expected, the low-energy band at 80 K is entirely due to the bulk of the crystal, as evidenced by the emission spectra measured in transmission geometry (Fig. S11†). Finally, we note that none of the PL spectra of the layered perovskites under study exhibit the broad emission at 1.4–2.1 eV often reported for this material class.^80,81^ However, since the latter is commonly attributed to below-gap bulk defect states, its presence is most likely highly dependent on the exact processing conditions of the single crystals.^80,81^

### Thermochromism, piezochromism, and phase stability

Due to the strong temperature dependence of the PL linewidth for the (F_2_)BTa-linked perovskites, these materials exhibit significant thermochromism, with the emission changing from orange at room temperature to bright green at 80 K (see emission photographs in Fig. 4a and graphical abstract). Such strong thermochromism is not observed for (PEA)_2_PbI_4_, since the dependence of the linewidth of the emission on temperature is not as strong as for the benzotriazole perovskites (*vide supra*). Additionally, it has been shown in the literature that, unlike benzotriazole perovskites, alkylammonium perovskites show phase transitions while cooling from RT to 80 K.^41^ Complementarily to this temperature-dependent behavior, we observe that the PL peak position of (BTa)_2_PbI_4_ and (F_2_BTa)_2_PbI_4_ is strongly dependent on the external pressure (Fig. S12†). For (BTa)_2_PbI_4_, the surface PL emission peak shifts from 550 nm (2.26 eV) at ambient pressure to 685 nm (1.82 eV) at 4.4 GPa. For (F_2_BTa)_2_PbI_4_, this is from 540 nm (2.30 eV) at ambient pressure to 620 nm (2.00 eV) at 5.0 GPa. The respective PL peak shifts are nearly linear at 100 meV GPa^−1^ and 60 meV GPa^−1^ (Fig. S13†). We tentatively attribute the weaker PL shift observed for (F_2_BTa)_2_PbI_4_ to the increased organic cohesive energy in this material in the form of intermolecular hydrogen bonding, which is not present in (BTa)_2_PbI_4_.^44^ The red-shifted bulk PL emission peak appears to be less sensitive to pressure changes and merges with the surface PL emission peak at about 1.6 GPa for both materials. We hypothesize that the crystallographic bulk-surface differences modelled in Fig. 3 are gradually eliminated with increasing pressure and that, at pressures above 1.6 GPa, the bulk and surface inorganic layers have nearly identical geometries. (PEA)_2_PbI_4_ also shows pressure-dependent behavior of its PL emission peak (97 meV GPa^−1^, Fig. S14†) but has been found to suffer from reversibility issues above 3.5 GPa.

**Fig. 5:**
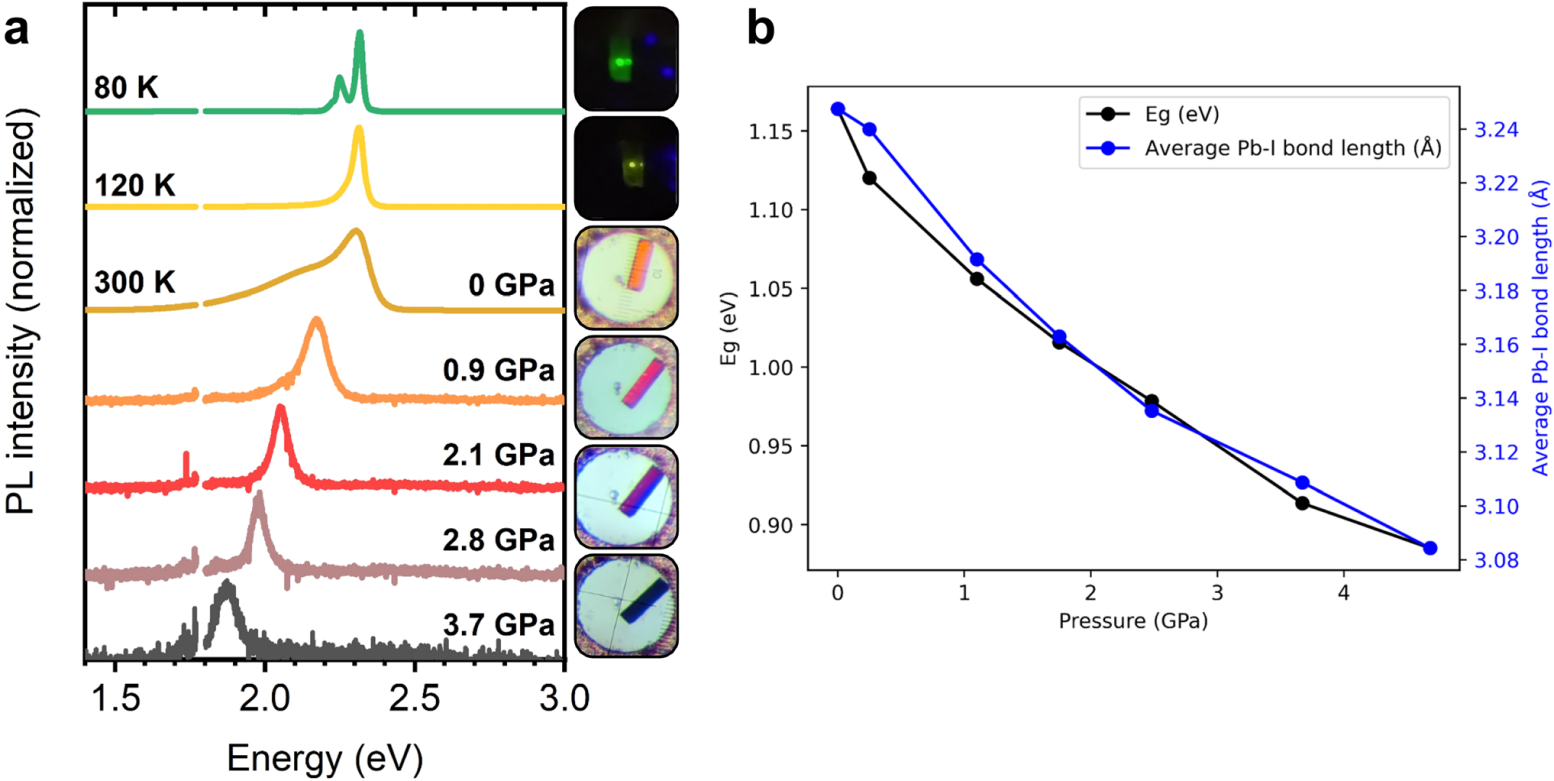
(a) PL emission shift of a (BTa)_2_PbI_4_ single crystal with temperature and with pressure, causing the crystal to change color. (b) Theoretically predicted band gap and average Pb–I bond length of (BTa)_2_PbI_4_ as a function of pressure, calculated at the PBE + SOC level of theory. Pressure-dependent structural models were obtained from atomic position optimization of crystal structures determined *via* single-crystal X-ray diffraction.

The pressure dependence of the PL emission peak is combined with the temperature dependence of the PL linewidth in Fig. 5a for (BTa)_2_PbI_4_ and in Fig. S15† for (F_2_BTa)_2_PbI_4_ to display the marked piezochromism and thermochromism in these materials. Both materials change color from orange to yellow to green upon cooling from ambient temperature to 80 K and from orange to red and brown to black upon compressing from ambient pressure to 5 GPa. In Fig. S16,† we verified that the PL spectra under standard conditions are the same, *i.e.* at 0 GPa in the pressure experiment and at 300 K in the temperature experiment. Furthermore, the PL changes are fully reversible in both temperature and pressure experiments. For the pressure experiment, this is shown in Fig. S17† and, as already mentioned above, is in contrast to (PEA)_2_PbI_4_. We have shown in a previous study that benzotriazole perovskites are also thermally stable at elevated temperatures up to 440 K.^44^ We also note that no phase transition occurs in (BTa)_2_PbI_4_ nor in (F_2_BTa)_2_PbI_4_ upon lowering the temperature from room temperature (293 K) to 100 K (Fig. S18, Tables S7 and S8†).

The pressure-dependent properties of the benzotriazole perovskites have also been explored theoretically. The structures obtained from *in situ* single-crystal X-ray diffraction during compression were relaxed with respect to atomic positions and, from these models, the electronic band gap was determined at the PBE level, including spin–orbit coupling. Including spin–orbit coupling is a crucial consideration for the accurate determination of optical and electronic properties in layered hybrid perovskites.^66,82,83^ For (BTa)_2_PbI_4_, the experimentally observed trend of decreasing band gap with increasing pressure is well-replicated by our calculations and is strongly correlated with the average Pb–I bond length in the crystal structure (Fig. 5b), which is consistent with more effective hybridization of the atomic orbitals and, therefore, with the broadening of the valence and conduction bands.^66^

## Conclusions

In this work, we revealed the impact of strong intermolecular interactions in the organic sublattice on the rigidity and optical properties of layered hybrid perovskites. We reported two benzotriazole-based perovskites with rigid organic layers, which we characterized with *in situ* single-crystal XRD under compression between 0 and 5 GPa and with an analysis of the isotropic displacement (*U*_eq_) parameters. At the same time, we found that the substantial increase in organic sublattice rigidity does not transfer to the inorganic sublattice. This results in an unprecedented combination of a rigid organic sublattice with a readily deformable inorganic sublattice. With PL emission spectroscopy and theoretical calculations, we exposed energetic and crystallographic differences between the crystal bulk and surface, leading to energy transfer processes. Additionally, with *in situ* PL spectroscopy during cooling, we found that inhomogeneous broadening dominates the emission profile for the benzotriazole perovskites while the profile of the reference perovskite (PEA)_2_PbI_4_ is dominated by homogeneous broadening. Further research is needed to fully elucidate the mechanism that is responsible for this atypical emission profile. Finally, we observed that the combination of the rigid benzotriazole organic sublattice with the deformable inorganic sublattice results in phase-stable perovskites with marked thermochromism and piezochromism, changing the crystal color between green, yellow, and orange with varying temperature and between orange, red, brown, and black with varying pressure, which opens up prospects for possible applications in temperature and/or pressure sensors.

## Author contributions

A. M., M. V. L., D. V., K. V., and W. T. M. V. G. conceptualized the research. A. M., M. V. L., M. D., P. B., P. L. M., and J. C. conducted the experiments. A. M., M. V. L., M. D., and P. B. wrote the manuscript. K. V. H., L. L., D. V., C. Q., D. B., S. P., K. V., and W. T. M. V. G. supervised the research activities. All authors contributed to the analysis of the data, reviewed the manuscript, and gave approval to the final version of the manuscript.

## Conflicts of interest

There are no conflicts of interest to declare.

## Supplementary Material

SC-016-D4SC06637E-s001

SC-016-D4SC06637E-s002

SC-016-D4SC06637E-s003

SC-016-D4SC06637E-s004

## Data Availability

Crystallographic data for this study has been deposited at the CCDC under 2365956–2365959, 2365961–2365964, 2365967–2365971, 2365975–2365987, and 2373937–2373938. These data can be obtained free of charge from The Cambridge Crystallographic Data Centre *via*https://www.ccdc.cam.ac.uk/structures. All other raw data are available from the authors upon reasonable request.

## References

[cit1] Dohner E. R., Hoke E. T., Karunadasa H. I. (2014). Self-assembly of broadband white-light emitters. J. Am. Chem. Soc..

[cit2] Tsai H., Nie W., Blancon J. C., Stoumpos C. C., Asadpour R., Harutyunyan B., Neukirch A. J., Verduzco R., Crochet J. J., Tretiak S., Pedesseau L., Even J., Alam M. A., Gupta G., Lou J., Ajayan P. M., Bedzyk M. J., Kanatzidis M. G. (2016). High-efficiency two-dimensional Ruddlesden-Popper perovskite solar cells. Nature.

[cit3] Li W., Sidhik S., Traore B., Asadpour R., Hou J., Zhang H., Fehr A., Essman J., Wang Y., Hoffman J. M., Spanopoulos I., Crochet J. J., Tsai E., Strzalka J., Katan C., Alam M. A., Kanatzidis M. G., Even J., Blancon J. C., Mohite A. D. (2022). Light-activated interlayer contraction in two-dimensional perovskites for high-efficiency solar cells. Nat. Nanotechnol..

[cit4] Sidhik S., Wang Y., De Siena M., Asadpour R., Torma A. J., Terlier T., Ho K., Li W., Puthirath A. B., Shuai X., Agrawal A., Traore B., Jones M., Giridharagopal R., Ajayan P. M., Strzalka J., Ginger D. S., Katan C., Alam M. A., Even J., Kanatzidis M. G., Mohite A. D. (2022). Deterministic fabrication of 3D/2D perovskite bilayer stacks for durable and efficient solar cells. Science.

[cit5] Qin C., Sandanayaka A. S. D., Zhao C., Matsushima T., Zhang D., Fujihara T., Adachi C. (2020). Stable room-temperature continuous-wave lasing in quasi-2D perovskite films. Nature.

[cit6] Zhang L., Sun C., He T., Jiang Y., Wei J., Huang Y., Yuan M. (2021). High-performance quasi-2D perovskite light-emitting diodes: from materials to devices. Light:Sci. Appl..

[cit7] Weerasinghe H. C., Macadam N., Kim J. E., Sutherland L. J., Angmo D., Ng L. W. T., Scully A. D., Glenn F., Chantler R., Chang N. L., Dehghanimadvar M., Shi L., Ho-Baillie A. W. Y., Egan R., Chesman A. S. R., Gao M., Jasieniak J. J., Hasan T., Vak D. (2024). The first demonstration of entirely roll-to-roll fabricated perovskite solar cell modules under ambient room conditions. Nat. Commun..

[cit8] Hong X., Ishihara T., Nurmikko A. V. (1992). Dielectric confinement effect on excitons in PbI_4_-based layered semiconductors. Phys. Rev. B:Condens. Matter Mater. Phys..

[cit9] Ishihara T., Takahashi J., Goto T. (1989). Exciton state in two-dimensional perovskite semiconductor (C_10_H_21_NH_3_)_2_PbI_4_. Solid State Commun..

[cit10] Koutselas I. B., Ducasse L., Papavassiliou G. C. (1996). Electronic properties of three- and low-dimensional semiconducting materials with Pb halide and Sn halide units. J. Phys.: Condens. Matter.

[cit11] Mitzi D. B., Chondroudis K., Kagan C. R. (2001). Organic–inorganic electronics. IBM J. Res. Dev..

[cit12] Katan C., Mercier N., Even J. (2019). Quantum and Dielectric Confinement Effects in Lower-Dimensional Hybrid Perovskite Semiconductors. Chem. Rev..

[cit13] Quarti C., Giorgi G., Katan C., Even J., Palummo M. (2023). Exciton Ground State Fine Structure and Excited States Landscape in Layered Halide Perovskites from Combined BSE Simulations and Symmetry Analysis. Adv. Opt. Mater..

[cit14] Du K. Z., Tu Q., Zhang X., Han Q., Liu J., Zauscher S., Mitzi D. B. (2017). Two-Dimensional Lead(ii) Halide-Based Hybrid Perovskites Templated by Acene Alkylamines: Crystal Structures, Optical Properties, and Piezoelectricity. Inorg. Chem..

[cit15] Marchenko E. I., Fateev S. A., Petrov A. A., Korolev V. V., Mitrofanov A., Petrov A. V., Goodilin E. A., Tarasov A. B. (2020). Database of Two-Dimensional Hybrid Perovskite Materials: Open-Access Collection of Crystal Structures, Band Gaps, and Atomic Partial Charges Predicted by Machine Learning. Chem. Mater..

[cit16] Quarti C., Marchal N., Beljonne D. (2018). Tuning the Optoelectronic Properties of Two-Dimensional Hybrid Perovskite Semiconductors with Alkyl Chain Spacers. J. Phys. Chem. Lett..

[cit17] Boeije Y., Van Gompel W. T. M., Zhang Y., Ghosh P., Zelewski S. J., Maufort A., Roose B., Ooi Z. Y., Chowdhury R., Devroey I., Lenaers S., Tew A., Dai L., Dey K., Salway H., Friend R. H., Sirringhaus H., Lutsen L., Vanderzande D., Rao A., Stranks S. D. (2023). Tailoring Interlayer Charge Transfer Dynamics in 2D Perovskites with Electroactive Spacer Molecules. J. Am. Chem. Soc..

[cit18] Liang A., Gao Y., Asadpour R., Wei Z., Finkenauer B. P., Jin L., Yang J., Wang K., Chen K., Liao P., Zhu C., Huang L., Boudouris B. W., Alam M. A., Dou L. (2021). Ligand-Driven Grain Engineering of High Mobility Two-Dimensional Perovskite Thin-Film Transistors. J. Am. Chem. Soc..

[cit19] Passarelli J. V., Fairfield D. J., Sather N. A., Hendricks M. P., Sai H., Stern C. L., Stupp S. I. (2018). Enhanced Out-of-Plane Conductivity and Photovoltaic Performance in *n* = 1 Layered Perovskites through Organic Cation Design. J. Am. Chem. Soc..

[cit20] Gelvez-Rueda M. C., Van Gompel W. T. M., Herckens R., Lutsen L., Vanderzande D., Grozema F. C. (2020). Inducing Charge Separation in Solid-State Two-Dimensional Hybrid Perovskites through the Incorporation of Organic Charge-Transfer Complexes. J. Phys. Chem. Lett..

[cit21] Van Landeghem M., Van Gompel W., Herckens R., Lutsen L., Vanderzande D., Van Doorslaer S., Goovaerts E. (2021). Light-Induced Charge Transfer in Two-Dimensional Hybrid Lead Halide Perovskites. J. Phys. Chem. C.

[cit22] Gao Y., Shi E., Deng S., Shiring S. B., Snaider J. M., Liang C., Yuan B., Song R., Janke S. M., Liebman-Pelaez A., Yoo P., Zeller M., Boudouris B. W., Liao P., Zhu C., Blum V., Yu Y., Savoie B. M., Huang L., Dou L. (2019). Molecular engineering of organic–inorganic hybrid perovskites quantum wells. Nat. Chem..

[cit23] Nussbaum S., Socie E., Yao L., Yum J.-H., Moser J.-E., Sivula K. (2022). Tuning Napththalenediimide Cations for Incorporation into Ruddlesden–Popper-Type Hybrid Perovskites. Chem. Mater..

[cit24] Feng Z., Liu X., Imaoka K., Ishii T., Tumen-Ulzii G., Tang X., Harrington G. F., Heinrich B., Ribierre J. C., Chamoreau L. M., Sosa Vargas L., Kreher D., Goushi K., Matsushima T., Zhou G., Mathevet F., Adachi C. (2023). Artificial p–n-like Junction Based on Pure 2D Organic–Inorganic Halide Perovskite Structure Having Naphthalene Diimide Acceptor Moieties. Adv. Opt. Mater..

[cit25] Dunlap-Shohl W. A., Barraza E. T., Barrette A., Dovletgeldi S., Findik G., Dirkes D. J., Liu C., Jana M. K., Blum V., You W., Gundogdu K., Stiff-Roberts A. D., Mitzi D. B. (2019). Tunable internal quantum well alignment in rationally designed oligomer-based perovskite films deposited by resonant infrared matrix-assisted pulsed laser evaporation. Mater. Horiz..

[cit26] Elshanawany M. M., Ricciardulli A. G., Saliba M., Wachtveitl J., Braun M. (2021). Mechanism of ultrafast energy transfer between the organic–inorganic layers in multiple-ring aromatic spacers for 2D perovskites. Nanoscale.

[cit27] Hu H., Meier F., Zhao D., Abe Y., Gao Y., Chen B., Salim T., Chia E. E. M., Qiao X., Deibel C., Lam Y. M. (2018). Efficient Room-Temperature Phosphorescence from Organic–Inorganic Hybrid Perovskites by Molecular Engineering. Adv. Mater..

[cit28] Yang S., Wu D., Gong W., Huang Q., Zhen H., Ling Q., Lin Z. (2018). Highly efficient room-temperature phosphorescence and afterglow luminescence from common organic fluorophores in 2D hybrid perovskites. Chem. Sci..

[cit29] Passarelli J. V., Mauck C. M., Winslow S. W., Perkinson C. F., Bard J. C., Sai H., Williams K. W., Narayanan A., Fairfield D. J., Hendricks M. P., Tisdale W. A., Stupp S. I. (2020). Tunable exciton binding energy in 2D hybrid layered perovskites through donor–acceptor interactions within the organic layer. Nat. Chem..

[cit30] Denis P. H., Mertens M., Van Gompel W. T. M., Maufort A., Mertens S., Wei Z., Van Landeghem M., Gielen S., Ruttens B., Deduytsche D., Detarvernier C., Lutsen L., Grozema F., Vandewal K., Vanderzande D. (2022). Quasi-2D Hybrid Perovskite Formation Using Benzothieno[3,2-*b*]Benzothiophene (BTBT) Ammonium Cations: Substantial Cesium Lead(II) Iodide Black Phase Stabilization. Adv. Opt. Mater..

[cit31] Xu Z., Lu D., Dong X., Chen M., Fu Q., Liu Y. (2021). Highly Efficient and Stable Dion-Jacobson Perovskite Solar Cells Enabled by Extended pi-Conjugation of Organic Spacer. Adv. Mater..

[cit32] Gao Y., Wei Z., Yoo P., Shi E., Zeller M., Zhu C., Liao P., Dou L. (2019). Highly Stable Lead-Free Perovskite Field-Effect Transistors Incorporating Linear pi-Conjugated Organic Ligands. J. Am. Chem. Soc..

[cit33] Li X., Hoffman J. M., Kanatzidis M. G. (2021). The 2D Halide Perovskite Rulebook: How the Spacer Influences Everything from the Structure to Optoelectronic Device Efficiency. Chem. Rev..

[cit34] Sun J., Wang K., Ma K., Park J. Y., Lin Z. Y., Savoie B. M., Dou L. (2023). Emerging Two-Dimensional Organic Semiconductor-Incorporated Perovskites horizontal line A Fascinating Family of Hybrid Electronic Materials. J. Am. Chem. Soc..

[cit35] Van Gompel W. T. M., Lutsen L., Vanderzande D. (2023). 2D and quasi-2D hybrid perovskites containing organic cations with an extended conjugated system: opportunities and challenges. J. Mater. Chem. C.

[cit36] Gong X., Voznyy O., Jain A., Liu W., Sabatini R., Piontkowski Z., Walters G., Bappi G., Nokhrin S., Bushuyev O., Yuan M., Comin R., McCamant D., Kelley S. O., Sargent E. H. (2018). Electron–phonon interaction in efficient perovskite blue emitters. Nat. Mater..

[cit37] Seitz M., Magdaleno A. J., Alcazar-Cano N., Melendez M., Lubbers T. J., Walraven S. W., Pakdel S., Prada E., Delgado-Buscalioni R., Prins F. (2020). Exciton diffusion in two-dimensional metal–halide perovskites. Nat. Commun..

[cit38] Liu Y., Zhou H., Ni Y., Guo J., Lu R., Li C., Guo X. (2023). Revealing stability origin of Dion-Jacobson 2D perovskites with different-rigidity organic cations. Joule.

[cit39] Zhao R., Sabatini R. P., Zhu T., Wang S., Morteza Najjarian A., Johnston A., Lough A. J., Hoogland S., Sargent E. H., Seferos D. S. (2021). Rigid Conjugated Diamine Templates for Stable Dion-Jacobson-Type Two-Dimensional Perovskites. J. Am. Chem. Soc..

[cit40] Gao C., Li R., Li Y., Wang R., Wang M., Gan Z., Bai L., Liu Y., Zhao K., Liu S. F., Cheng Y., Huang W. (2019). Direct–Indirect Transition of Pressurized Two-Dimensional Halide Perovskite: Role of Benzene Ring Stack Ordering. J. Phys. Chem. Lett..

[cit41] Mauck C. M., France-Lanord A., Hernandez Oendra A. C., Dahod N. S., Grossman J. C., Tisdale W. A. (2019). Inorganic Cage Motion Dominates Excited-State Dynamics in 2D-Layered Perovskites (C_*x*_H_2*x*+1_NH_3_)_2_PbI_4_ (*x* = 4–9). J. Phys. Chem. C.

[cit42] Qin Y., Lv Z., Chen S., Li W., Wu X., Ye L., Li N., Lu P. (2019). Tuning Pressure-Induced Phase Transitions, Amorphization, and Excitonic Emissions of 2D Hybrid Perovskites *via* Varying Organic Amine Cations. J. Phys. Chem. C.

[cit43] Caiazzo A., Maufort A., van Gorkom B. T., Remmerswaal W. H. M., Orri J. F., Li J., Wang J., van Gompel W. T. M., Van Hecke K., Kusch G., Oliver R. A., Ducati C., Lutsen L., Wienk M. M., Stranks S. D., Vanderzande D., Janssen R. A. J. (2023). 3D Perovskite Passivation with a Benzotriazole-Based 2D Interlayer for High-Efficiency Solar Cells. ACS Appl. Energy Mater..

[cit44] Maufort A., Cerda J., Van Hecke K., Deduytsche D., Verding A., Ruttens B., Li W., Detavernier C., Lutsen L., Quarti C., Vanderzande D., Beljonne D., Van Gompel W. T. M. (2024). Elucidating the Non-Covalent Interactions that Trigger Interdigitation in Lead-Halide Layered Hybrid Perovskites. Inorg. Chem..

[cit45] Liu S., Sun S., Gan C. K., Del Aguila A. G., Fang Y., Xing J., Do T. T. H., White T. J., Li H., Huang W., Xiong Q. (2019). Manipulating efficient light emission in two-dimensional perovskite crystals by pressure-induced anisotropic deformation. Sci. Adv..

[cit46] Yin T., Liu B., Yan J., Fang Y., Chen M., Chong W. K., Jiang S., Kuo J. L., Fang J., Liang P., Wei S., Loh K. P., Sum T. C., White T. J., Shen Z. X. (2019). Pressure-Engineered Structural and Optical Properties of Two-Dimensional (C_(4)_H_(9)_NH_(3)_)_(2)_PbI_(4)_ Perovskite Exfoliated nm-Thin Flakes. J. Am. Chem. Soc..

[cit47] Zhang L., Wang K., Lin Y., Zou B. (2020). Pressure Effects on the Electronic and Optical Properties in Low-Dimensional Metal Halide Perovskites. J. Phys. Chem. Lett..

[cit48] Muscarella L. A., Ducinskas A., Dankl M., Andrzejewski M., Casati N. P. M., Rothlisberger U., Maier J., Graetzel M., Ehrler B., Milic J. V. (2022). Reversible Pressure-Dependent Mechanochromism of Dion–Jacobson and Ruddlesden–Popper Layered Hybrid Perovskites. Adv. Mater..

[cit49] Azeem M., Qin Y., Li Z.-G., Li W. (2021). Cooperative B-site octahedral tilting, distortion and A-site conformational change induced phase transitions of a 2D lead halide perovskite. Mater. Chem. Front..

[cit50] Baur W. H. (1974). The geometry of polyhedral distortions. Predictive relationships for the phosphate group. Acta Crystallogr..

[cit51] Billing D. G., Lemmerer A. (2007). Synthesis, characterization and phase transitions in the inorganic–organic layered perovskite-type hybrids [(C_*n*_H_2*n*+1_NH_3_)_2_PbI_4_], *n* = 4, 5 and 6. Acta Crystallogr., Sect. B:Struct. Sci..

[cit52] Straus D. B., Iotov N., Gau M. R., Zhao Q., Carroll P. J., Kagan C. R. (2019). Longer Cations Increase Energetic Disorder in Excitonic 2D Hybrid Perovskites. J. Phys. Chem. Lett..

[cit53] Fridriksson M. B., van der Meer N., de Haas J., Grozema F. C. (2020). Tuning the Structural Rigidity of Two-Dimensional Ruddlesden–Popper Perovskites through the Organic Cation. J. Phys. Chem. C.

[cit54] Yuan Y., Liu X. F., Ma X., Wang X., Li X., Xiao J., Li X., Zhang H. L., Wang L. (2019). Large Band Gap Narrowing and Prolonged Carrier Lifetime of (C_(4)_H_(9)_NH_(3)_)_(2)_PbI_(4)_ under High Pressure. Adv. Sci..

[cit55] Feng G., Qin Y., Ran C., Ji L., Dong L., Li W. (2018). Structural evolution and photoluminescence properties of a 2D hybrid perovskite under pressure. APL Mater..

[cit56] Du Q., Zhu C., Yin Z., Na G., Cheng C., Han Y., Liu N., Niu X., Zhou H., Chen H., Zhang L., Jin S., Chen Q. (2020). Stacking Effects on Electron–Phonon Coupling in Layered Hybrid Perovskites *via* Microstrain Manipulation. ACS Nano.

[cit57] Moral R. F., Germino J. C., Bonato L. G., Almeida D. B., Therézio E. M., Atvars T. D. Z., Stranks S. D., Nome R. A., Nogueira A. F. (2020). Influence of the Vibrational Modes from the Organic Moieties in 2D Lead Halides on Excitonic Recombination and Phase Transition. Adv. Opt. Mater..

[cit58] Sheikh T., Shinde A., Mahamuni S., Nag A. (2019). Dual excitonic emissions and structural phase transition of octylammonium lead iodide 2D layered perovskite single crystal. Mater. Res. Express.

[cit59] Wang Y., He C., Tan Q., Tang Z., Huang L., Liu L., Yin J., Jiang Y., Wang X., Pan A. (2023). Exciton–phonon coupling in two-dimensional layered (BA)_(2)_PbI_(4)_ perovskite microplates. RSC Adv..

[cit60] Gan Z., Wen X., Chen W., Zhou C., Yang S., Cao G., Ghiggino K. P., Zhang H., Jia B. (2019). The Dominant Energy Transport Pathway in Halide Perovskites: Photon Recycling or Carrier Diffusion?. Adv. Energy Mater..

[cit61] Kutkan S., Dhanabalan B., Lin M. L., Tan P. H., Schleusener A., Arciniegas M. P., Krahne R. (2023). Impact of the organic cation on the band-edge emission of two-dimensional lead-bromide perovskites. Nanoscale.

[cit62] Lyu D., Miao Y., Li B., Xiao Z., Wu X., Hu X., Jiang X.-F., Xu Q.-H. (2021). Dual Blue Emission in Ruddlesden–Popper Lead-Bromide Perovskites Induced by Photon Recycling. J. Phys. Chem. C.

[cit63] Jin T., Liu Z., Luo J., Yuan J. H., Wang H., Xie Z., Pan W., Wu H., Xue K. H., Liu L., Hu Z., Zheng Z., Tang J., Niu G. (2023). Self-wavelength shifting in two-dimensional perovskite for sensitive and fast gamma-ray detection. Nat. Commun..

[cit64] Knutson J. L., Martin J. D., Mitzi D. B. (2005). Tuning the band gap in hybrid tin iodide perovskite semiconductors using structural templating. Inorg. Chem..

[cit65] Sourisseau S., Louvain N., Bi W., Mercier N., Rondeau D., Boucher F., Buzaré J.-Y., Legein C. (2007). Reduced Band Gap Hybrid Perovskites Resulting from Combined Hydrogen and Halogen Bonding at the Organic–Inorganic Interface. Chem. Mater..

[cit66] Pedesseau L., Sapori D., Traore B., Robles R., Fang H. H., Loi M. A., Tsai H., Nie W., Blancon J. C., Neukirch A., Tretiak S., Mohite A. D., Katan C., Even J., Kepenekian M. (2016). Advances and Promises of Layered Halide Hybrid Perovskite Semiconductors. ACS Nano.

[cit67] Sheikh T., Shinde A., Mahamuni S., Nag A. (2018). Possible Dual Bandgap in (C_4_H_9_NH_3_)_2_PbI_4_ 2D Layered Perovskite: Single-Crystal and Exfoliated Few-Layer. ACS Energy Lett..

[cit68] Rudin S., Reinecke T. L., Segall B. (1990). Temperature-dependent exciton linewidths in semiconductors. Phys. Rev. B:Condens. Matter Mater. Phys..

[cit69] Wright A. D., Verdi C., Milot R. L., Eperon G. E., Perez-Osorio M. A., Snaith H. J., Giustino F., Johnston M. B., Herz L. M. (2016). Electron–phonon coupling in hybrid lead halide perovskites. Nat. Commun..

[cit70] Tekelenburg E. K., Kahmann S., Kamminga M. E., Blake G. R., Loi M. A. (2021). Elucidating the Structure and Photophysics of Layered Perovskites through Cation Fluorination. Adv. Opt. Mater..

[cit71] Song J., Feng X., Wei H., Yang B. (2022). Supramolecular Interactions of Flexible 2D Perovskite in Microstrain Releasing and Optoelectronic Properties Recovery. Adv. Funct. Mater..

[cit72] Zhang B., Zheng T., You J., Ma C., Liu Y., Zhang L., Xi J., Dong G., Liu M., Liu S. F. (2023). Electron–Phonon Coupling Suppression by Enhanced Lattice Rigidity in 2D Perovskite Single Crystals for High-Performance X-Ray Detection. Adv. Mater..

[cit73] Perez C. M., Ghosh D., Prezhdo O., Nie W., Tretiak S., Neukirch A. (2022). Point Defects in Two-Dimensional Ruddlesden–Popper Perovskites Explored with *Ab Initio* Calculations. J. Phys. Chem. Lett..

[cit74] Ni L., Huynh U., Cheminal A., Thomas T. H., Shivanna R., Hinrichsen T. F., Ahmad S., Sadhanala A., Rao A. (2017). Real-Time Observation of Exciton–Phonon Coupling Dynamics in Self-Assembled Hybrid Perovskite Quantum Wells. ACS Nano.

[cit75] Mukamel S. , Principles of Nonlinear Optical Spectroscopy, Oxford University Press, 1995

[cit76] Straus D. B., Hurtado Parra S., Iotov N., Gebhardt J., Rappe A. M., Subotnik J. E., Kikkawa J. M., Kagan C. R. (2016). Direct Observation of Electron–Phonon Coupling and Slow Vibrational Relaxation in Organic–Inorganic Hybrid Perovskites. J. Am. Chem. Soc..

[cit77] Straus D. B., Hurtado Parra S., Iotov N., Zhao Q., Gau M. R., Carroll P. J., Kikkawa J. M., Kagan C. R. (2020). Tailoring Hot Exciton Dynamics in 2D Hybrid Perovskites through Cation Modification. ACS Nano.

[cit78] Urban J. M., Chehade G., Dyksik M., Menahem M., Surrente A., Trippe-Allard G., Maude D. K., Garrot D., Yaffe O., Deleporte E., Plochocka P., Baranowski M. (2020). Revealing Excitonic Phonon Coupling in (PEA)(2)(MA)(*n* − 1)Pb(*n*)I(3*n* + 1) 2D Layered Perovskites. J. Phys. Chem. Lett..

[cit79] Fu J., Bian T., Yin J., Feng M., Xu Q., Wang Y., Sum T. C. (2024). Organic and inorganic sublattice coupling in two-dimensional lead halide perovskites. Nat. Commun..

[cit80] Kahmann S., Meggiolaro D., Gregori L., Tekelenburg E. K., Pitaro M., Stranks S. D., De Angelis F., Loi M. A. (2022). The Origin of Broad Emission in 〈100〉 Two-Dimensional Perovskites: Extrinsic *vs.* Intrinsic Processes. ACS Energy Lett..

[cit81] Kahmann S., Tekelenburg E. K., Duim H., Kamminga M. E., Loi M. A. (2020). Extrinsic nature of the broad photoluminescence in lead iodide-based Ruddlesden–Popper perovskites. Nat. Commun..

[cit82] Even J., Pedesseau L., Dupertuis M. A., Jancu J. M., Katan C. (2012). Electronic model for self-assembled hybrid organic/perovskite semiconductors: Reverse band edge electronic states ordering and spin–orbit coupling. Phys. Rev. B:Condens. Matter Mater. Phys..

[cit83] Even J., Pedesseau L., Jancu J.-M., Katan C. (2013). Importance of Spin–Orbit Coupling in Hybrid Organic/Inorganic Perovskites for Photovoltaic Applications. J. Phys. Chem. Lett..

