# Supplementary material for: Structural rigidity, thermochromism and piezochromism of layered hybrid perovskites containing an interdigitated organic bilayer
Source: Chem Sci. 2025 Feb 24;16(13):5662–75. doi: 10.1039/d4sc06637e (PMC11874244; doi:10.1039/d4sc06637e)
Supplement: SC-016-D4SC06637E-s001 [file SC-016-D4SC06637E-s001.pdf]

Supplementary Information (SI) for Chemical Science.
This journal is © The Royal Society of Chemistry 2025

Supporting information

Structural rigidity, thermochromism and piezochromism of layered hybrid perovskites containing an interdigitated organic bilayer

1. Synthesis of single crystals

The synthesis of single crystals of $(PEA)_2PbI_4$, $(BTa)_2PbI_4$, and $(F_2BTa)_2PbI_4$ has been reported in earlier work by the authors,[1, 2] following the solvent conversion-induced crystallization method reported by Fateev et al.[3] Briefly, Stoichiometric amounts of the organic halide and lead halide were dissolved in a mixture of propylene carbonate (PC), water, and HBr or HI (48% resp. 57% in water). HI was extracted three times with a 9:1 v/v mixture of chloroform and tri-*n*-butyl phosphate before use. The mixtures were stirred at 50 °C until a clear solution was obtained, were filtered through a syringe filter into a base-bath cleaned glass vial, and were then heated to their crystallization temperature. The lid of the vial was perforated with a needle to enable CO_2 to escape throughout the solvent degradation process. Precursor concentrations, solvent mixtures and crystallization temperatures are summarized in **Table S1**.

Table S1. Experimental conditions for solvent conversion-induced crystallization.

	**Organic halide**	**Lead halide**	**Solvents**	**Crystallization temperature**
**$(BTa)_2PbI_4$**	0.29 M BTaI	0.15 M PbI_2	$PC/H_2O/HI$ 1:2:0.2 molar ratio	50 °C (24 h)
**$(F_2BTa)_2PbI_4$**	0.60 M F_2BTaI	0.30 M PbI_2		40 °C (48 h)
**$(PEA)_2PbI_4$**	1.25 M PEAI	0.63 M PbI_2		50 °C (24 h)

2. XRD characterization

In previous work,[1,2] X-ray intensity data of $(BTa)_2PbI_4$ and $(F_2BTa)_2PbI_4$ single crystals has been collected at 100 K. In this work, X-ray intensity data of these single crystals was collected at 293 K, on a Rigaku Oxford Diffraction Supernova Dual Source (Cu at zero) diffractometer equipped with an Atlas CCD detector using ω scans and CuK_α (λ = 1.54184 Å) radiation. The images were interpreted and integrated with the program CrysAlisPro (Rigaku Oxford Diffraction **2022**, CrysAlisPro Software system, version 1.171.42.49, Rigaku Corporation, Oxford, UK.). Using Olex2,[4] the structures were solved by direct methods using the ShelXT structure solution program and refined by full-matrix least-squares on F^2 using the ShelXL program package.[5, 6] Non-hydrogen atoms were anisotropically refined, with the hydrogen atoms in the riding mode and isotropic temperature factors fixed at 1.2 times U(eq) of the parent atoms (1.5 times for methyl groups).

The high-pressure single-crystal X-ray diffraction measurements were carried out at 300 K on one single crystal of $(BTa)_2PbI_4$ and $(F_2BTa)_2PbI_4$. Crystals were loaded in a Membrane Diamond Anvil Cell (MDAC) in a stainless-steel gasket pre-indented at 124 µm with small ruby balls for the in-situ measurement of pressure by ruby luminescence (PRL) technique.[7] Daphnee oil 7474 was used as pressure-transmitting media allowing a hydrostatic pressure till 3.7 GPa.[8] X-ray diffraction was measured on a Bruker D8 Venture diffractometer equipped with a molybdenum microfocus X-ray tube (MoK_α radiation, λ = 0.71073 Å) with mirror optics as a monochromator, and a PHOTON III CMOS detector of size 10 x 14 cm, a short collimator and long beamstop were used as described by Vijayakumar-Symala et al.[9] Optical centering perpendicular to the MDAC axis was done using the camera of the PRL device while the centering along the MDAC axis was performed according to the procedure described by Dawson et al.[10] Data integration and reduction were carried out using the SAINT program after excluding the shadowed regions with a dynamic mask. A half-aperture angle of 43° is used in the Diamond Anvil Cell section of the integration interface of the software. An empirical absorption correction based on the intensities using SADABS[11] was used. The structure was solved using the SHELXT program and refined using the SHELXL program[5, 6] in the Olex2-1.3 suite.[4] For all the datasets, only the heavier atoms (Pb, I and F in F_2Bta_2PbI4) could be refined anisotropically, C and N atoms were refined with isotropic thermal motion parameters, H atoms were placed at the calculated values (HFIX command). Completeness is around 50% for $(BTa)_2PbI_4$ and 40% for $(F_2BTa)_2PbI_4$ up to a resolution of 0.83 Å, see **Table S2** for more details. Pressure points were measured in an increasing and decreasing manner (see **Table S2** for details) showing no hysteresis in the volume for both samples, highlighting that only elastic reversible changes occur in our experiments. For both samples, the evolution of unit cell volume as a function of pressure was measured and points below the hydrostatic limit were used to fit a 3rd order Birch-Murnaghan Equation of State (EoS)[12] using the EosFit7-GUI software.[13]

Both compounds retain their orthorhombic Pnma space group under pressure. No phase transitions were observed in the pressure range of the experiment.

Table S2. Lattice parameters and refinement parameters for the *in situ* single-crystal XRD measurements under pressure.

$(BTa)_2PbI_4$

p (GPa)	SG	*a* (Å)	*b* (Å)	*c* (Å)	*V* (Å^3)	*R*int	Compl. (0.83Å)	*d* Min Å	Redud.	I/sigma	*N*refl/*N*para	R_1 (%)	wR_2(%)	GooF
0.00(2)	Pnma	12.9311(9)	29.900(2)	6.6301(8)	2563.4(4)	5.22%	52.75%	0.8011		35.9229	16.5	2.36%	5.22%	1.039
0.26(2)	Pnma	12.8539(6)	29.6936(13)	6.5896(6)	2515.1(3)	4.98%	52.20%	0.8025	11.08	35.2015	16	2.28%	5.33%	1.089
0.62(2)	Pnma	12.7554(5)	29.3983(12)	6.5298(5)	2448.6(2)	4.95%	52.70%	0.8036	11.09	36.3404	15.7	2.32%	5.61%	1.135
1.10(2)	Pnma	12.6467(5)	29.0301(12)	6.4684(5)	2374.8(2)	4.95%	52.60%	0.7999	10.88	37.1984	15.2	2.25%	4.69%	1.162
1.75(2)	Pnma	12.5209(6)	28.5807(14)	6.4040(6)	2291.7(3)	4.81%	52.90%	0.8033	10.97	37.0949	14.7	2.22%	4.95%	1.083
2.25(3)	Pnma	12.4439(5)	28.3074(11)	6.3671(5)	2242.8(2)	4.76%	52.40%	0.8013	10.82	38.211	14.3	2.40%	5.54%	1.166
3.13(5)	Pnma	12.3379(5)	27.9425(10)	6.3129(5)	2176.4(2)	4.74%	52.30%	0.8065	11.01	36.4573	13.9	2.21%	4.69%	1.183
3.67(5)	Pnma	12.2754(5)	27.734(11)	6.2807(5)	2138.2(2)	4.66%	52.60%	0.8048	10.9	36.0349	13.7	2.12%	4.15%	1.112
4.27(4)	Pnma	12.2581(6)	27.5707(12)	6.2184(5)	2101.6(2)	4.03%	52.60%	0.8039	11	46.1296	13.7	2.02%	4.16%	1.099
4.69(4)	Pnma	12.2480(7)	27.4906(15)	6.1820(6)	2081.5(3)	3.93%	52.70%	0.8016		49.4629	13.5	2.24%	4.82%	1.114
2.48(6)	Pnma	12.4242(5)	28.2364(11)	6.3612(5)	2231.6(2)	4.42%	52.20%	0.8019	10.89	39.4572	14.3	2.04%	3.87%	1.171
1.23(4)	Pnma	12.6137(6)	28.9129(13)	6.4557(5)	2354.4(2)	4.18%	49.40%	0.8039	10.76	41.7263	14.1	2.09%	4.64%	1.17
0.14(3)	Pnma	12.8828(18)	29.781(4)	6.6079(16)	2535.2(8)	4.76%	41.50%	0.8017	10.95	43.1262	12.8	2.21%	5.56%	1.079

$(F_2BTa)_2PbI_4$

p (GPa)	SG	*a* (Å)	*b* (Å)	*c* (Å)	*V* (Å^3)	*R*int (%)	completness	*d* Min (Å)	Redud.	I/sigma	*N*refl/*N*para	R_1 (%)	wR_2(%)	GooF
0.00(2)	Pnma	12.9388(4)	30.291(2)	6.6444(4)	2604.1(2)	3.62%	40.60%	0.81		76.5	10.6	1.70%	3.89%	1.102
0.20(5)	Pnma	12.9028(5)	30.077(3)	6.6218(3)	2569.8(3)	3.84%	40.40%	0.81	10.53	66	10.3	1.70%	3.90%	1.113
0.60(2)	Pnma	12.8444(5)	29.723(3)	6.5815(3)	2512.6(3)	3.87%	40.80%	0.81	10.39	60.2	10.2	1.67%	3.87%	1.103
1.10(2)	Pnma	12.75825(5)	29.234(3)	6.5183(3)	2431.1(2)	3.98%	40.90%	0.8	12.07	54.9	9.94	1.66%	3.50%	1.118
1.60(2)	Pnma	12.6990(5)	28.928(2)	6.4727(3)	2377.8(2)	3.73%	41.10%	0.81	10.29	54.6	10.3	1.68%	3.94%	1.153
2.22(2)	Pnma	12.6346(5)	28.617(2)	6.4221(3)	2322.0(2)	3.66%	41.40%	0.81	10.27	59.3	9.62	1.51%	3.19%	1.154
2.83(3)	Pnma	12.5704(5)	28.337(2)	6.3725(3)	2269.9(2)	3.65%	41.90%	0.8	10.13	59.3	9.96	1.58%	3.75%	1.158
3.40(2)	Pnma	12.5155(5)	28.112(2)	6.3307(2)	2227.3(2)	3.59%	42.00%	0.8	9.99	58.8	10.4	1.70%	3.94%	1.204
4.33(5)	Pnma	12.5029(7)	27.709(3)	6.2997(4)	2182.5(3)	3.55%	42.10%	0.8	9.53	63.7	9.15	1.85%	4.10%	1.157
4.81(2)	Pnma	12.5017(9)	27.526(4)	6.2872(4)	2163.6(4)	3.60%	42.40%	0.81	9.25	65.2	9.06	2.23%	4.59%	1.191
3.90(2)	Pnma	12.4841(6)	27.931(2)	6.2972(3)	2195.8(2)	3.30%	41.30%	0.8	9.66	67.6	9.05	1.59%	3.58%	1.117
1.61(4)	Pnma	12.6989(10)	28.912(4)	6.4700(5)	2375.5(4)	3.53%	41.70%	0.81	10.34	61.7	9.87	1.51%	3.55%	1.146
0.10(2)	Pnma	12.9362(8)	30.258(3)	6.6404(4)	2599.2(4)	3.70%	52.10%	0.8	7.92	41	13.5	2.18%	5.06%	1.057

3. PL characterization

Photoluminescence (PL) experiments at ambient pressure were performed under excitation with a 405 nm pulsed diode laser (Thorlabs, NPL41C, 129 ns pulse length and 128 nJ per pulse). The single crystals were suspended in an N_2 gas exchange cryostat (Oxford Instruments) to allow for variable-temperature experiments and to avoid photo-oxidation of the crystals during laser exposure. The cryostat had optical access from two opposite sides, enabling excitation by a focused laser beam either from the front or back of the crystal with respect to the collecting and collimating optics in front of the spectrograph entrance. In the main text, these different measurement geometries are denoted by 'confocal' and 'transmission', respectively. The collected PL emission was then diffracted in a grating spectrograph (Andor, Kymera 328i-D2-SIL) and registered by a linear CCD detector (Andor, iDus 416-LDC-DD).

Time-resolved photoluminescence (TRPL) experiments were performed under excitation with the 405 nm pulsed diode laser in the same setup. After diffraction in the spectrograph, the PL emission was registered by an intensified CCD detector (Andor, Istar DH320T-18F-74) which allows for the time-gated acquisition of PL signals in a time range of ns up to 100's of ms after the excitation pulse.

Unwanted laser reflections were eliminated from the spectra by means of a 405 nm notch filter in front of the spectrograph entrance. All spectra were corrected for the spectral response of the setup determined using a calibrated light source (Avantes, AvaLight-HAL-CAL-Mini).

High-pressure PL measurements have been performed on single crystals using a home-built experimental setup. For constituency of the results, exact same conditions as for the high-pressure crystallographic experiment have been used. A single crystal was loaded in a Membrane Diamond Anvil Cell (MDAC) in a stainless-steel gasket pre-indented at 124 µm with small ruby balls for the in-situ measurement of pressure by ruby luminescence technique. Daphnee oil 7474 was used as pressure transmitting media. The filled MDAC was mounted on the goniometer of a STOE STADIVARI diffractometer equipped with the PL setup. The perfect control of the MDAC orientation is provided by the goniometer. For PL measurements, the excitation source was a 405 nm CW diode laser. The PL signal was collected through a beam expander (Thorlabs, GBE20-A) and injected into a multimode bifurcated optical fiber bundle. A 420 nm low pass color filter was placed between the MDAC and the beam expander to cut the 405 nm excitation source. One branch of the optical fiber was connected to a HR4000 Ocean Optics spectrometer for ruby luminescence determination of the pressure inside the MDAC, while the other branch of the optical fiber was connected to a USB4000 Ocean Optics spectrometer for PL measurements.

4. Computational details

Periodic plane-wave density functional theory simulations of the investigated structures were performed using the QuantumEspresso package, employing the PBE exchange-correlation functional.[14-17] Core electrons were modeled using norm-conserving, fully relativistic PBE pseudopotentials from the pseudodojo database.[18] Plane-wave expansions of the electron density were constructed using kinetic energy and charge density cutoffs of 50 and 200 Rydberg, respectively.

Both calculations on the slab model (Figure 3) and on the crystal structures under pressure (Figure 5) underwent to full geometry optimization of the atomic positions, with fixed cell parameters. For the latter, we fixed the space group to the one evinced from XRD measurements, while for the former we only imposed a mirror plane symmetry with respect to the stacking direction, to avoid spurious electrostatic interactions with the periodic replica. In view of the different size of the lattice parameters for the slab and for the bulk models, we used different sampling of the reciprocal space. Specifically, we employed automatic 2x2x1 and 4x2x4 Monkhorst-Pack sampling of the Brillouin zone,[19] the less dense sampling being associated with the reciprocal lattice parameter associated with the (longer) plane stacking direction. Geometric optimization of atomic positions for the systems was performed using a convergence criterion of $\Delta E = 10^{-7}$ Rydberg of the self-consistent field energy complemented with Grimme's D3 dispersion corrections.[20]

Upon optimization, determinations of electronic states were performed using a more stringent convergence criterion of $\Delta E = 10^{-8}$ Rydberg, in non-collinear spin frame, including spin-orbit coupling.

5. Supporting crystallographic data

Crystallographic data of $(BA)_2PbI_4$, $(HA)_2PbI_4$, $(PEA)_2PbI_4$, $(NEA)_2PbI_4$, $(BTa)_2PbI_4$, and $(F_2BTa)_2PbI_4$ are provided free of charge by the joint Cambridge Crystallographic Data Centre and Fachinformationszentrum Karlsruhe *via* www.ccdc.cam.ac.uk/structures.

$(BA)_2PbI_4$ (293 K): CCDC 665690, reported by Billing and Lemmerer.[21]

$(HA)_2PbI_4$ (293 K): CCDC 665695, reported by Billing and Lemmerer.[21]

$(PEA)_2PbI_4$ (296 K): CCDC 1542461 & 1977183, reported by Du et al.[22] and Straus et al,[23] respectively.

$(NEA)_2PbI_4$ (296 K): CCDC 1542463, reported by Du et al.[22]

$(BTa)_2PbI_4$ (293 K): CCDC 2373937, this work.

$(F_2BTa)_2PbI_4$ (293 K): CCDC 2373938, this work.

$(PEA)_2PbI_4$ (100 K): CCDC 1977184 & 1515121, reported by Straus et al.[23] and Ma et al,[24] respectively.

$(BTa)_2PbI_4$ (100 K): CCDC 2228611, reported by Caiazzo et al.[2]

$(F_2BTa)_2PbI_4$ (100 K): CCDC 2228612, reported by Caiazzo et al.[2]

Table S3. CCDC codes of $(BTa)_2PbI_4$ and $(F_2BTa)_2PbI_4$ at normal and elevated pressure. Each time, the first ten crystal structures were measured while building up the pressure; the last three measurements while releasing the pressure.

**$(BTa)_2PbI_4$**		**$(F_2BTa)_2PbI_4$**	
Pressure (GPa)	CCDC code	Pressure (GPa)	CCDC code
0	2365983	0	2365956
0.26	2365980	0.20	2365957
0.62	2365976	0.60	2365958
1.10	2365977	1.10	2365959
1.75	2365986	1.60	2365961
2.25	2365981	2.22	2365962
3.13	2365978	2.83	2365963
3.67	2365985	3.40	2365964
4.27	2365982	4.33	2365967
4.69	2365975	4.81	2365968
2.48	2365987	3.90	2365969
1.23	2365979	1.61	2365970
0.14	2365984	0.10	2365971

6. Supporting experimental results

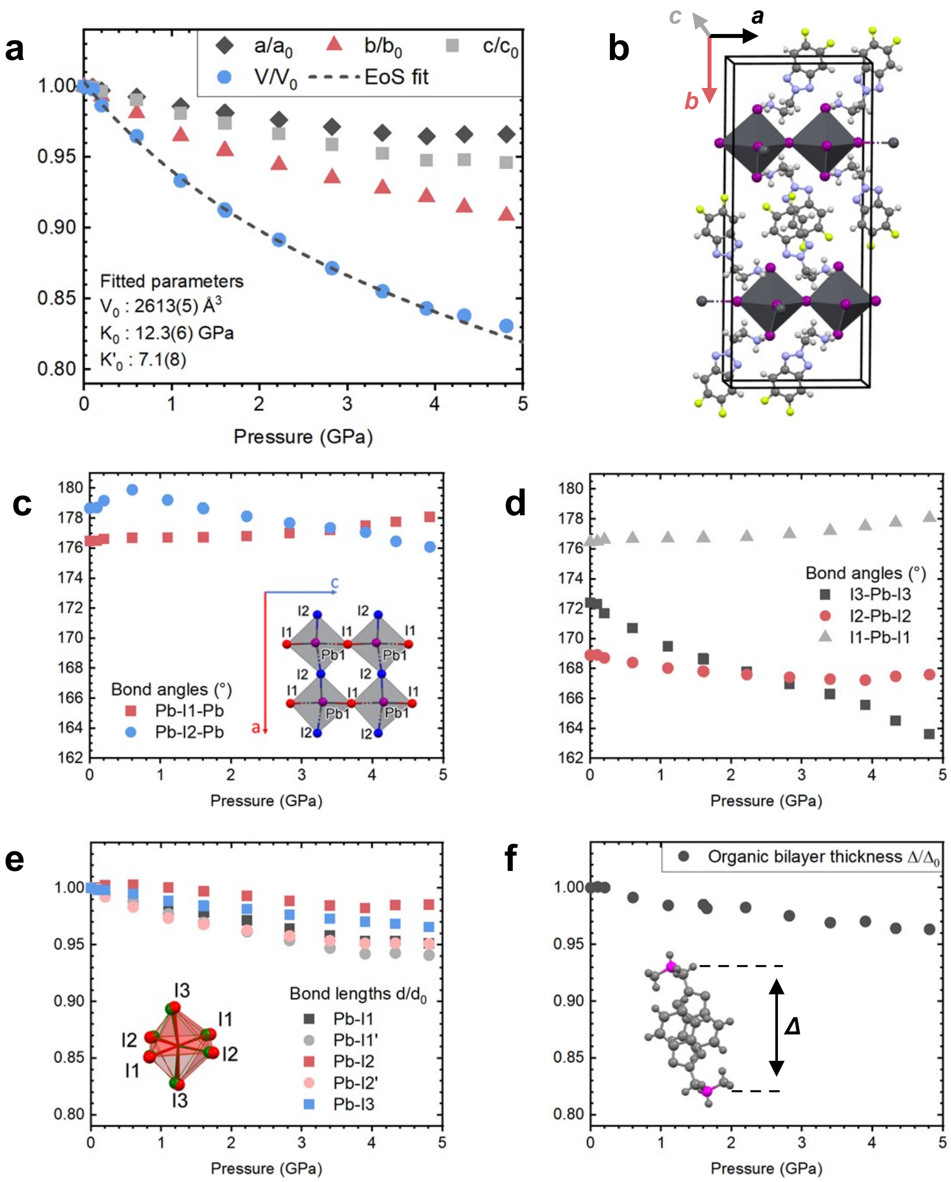

Figure S1. Evolution of the crystal structure of $(F_2BTa)_2PbI_4$ in the 0–5 GPa pressure domain. (a) lattice parameters and cell volume (which are defined in (b)), (c) Pb-I-Pb bond angles, (d) I-Pb-I bond angles, (e) Pb-I bond lengths, and (f) organic bilayer thickness.

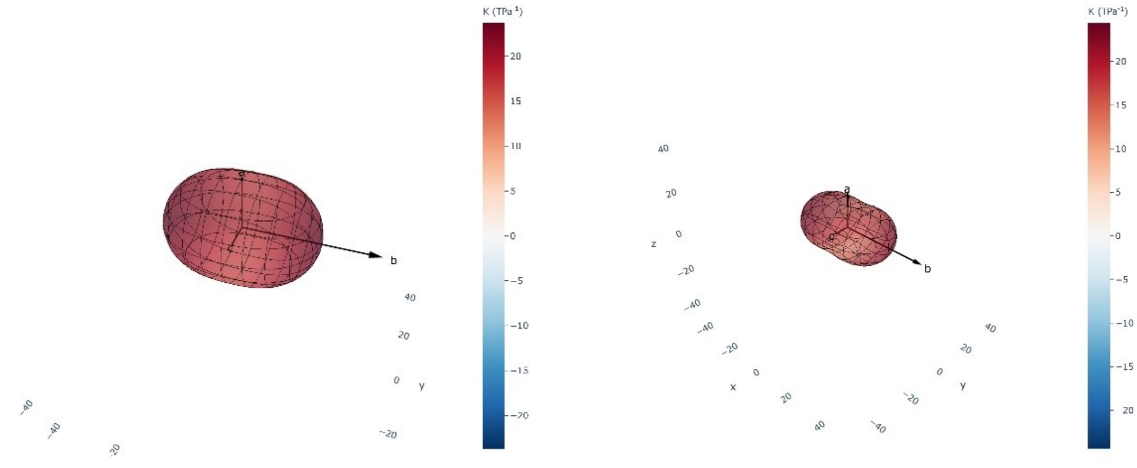

a **b**

Figure S2. Compressibility indicatrix for (a) $(BTa)_2PbI_4$ and (b) $(F_2BTa)_2PbI_4$. Compressibility tensors were drawn using the PASCAL (Principal Axis Strain Calculator, https://www.pascalapp.co.uk/) web-tool, showing that, for both compounds, the more compressible axis is *b*, which is the direction of the inorganic/organic layer stacking. The linear compressibilities (obtained when the pressure is hydrostatic below 3.7 GPa) of $(BTa)_2PbI_4$ are K_{X1} = 23.7395 TPa^{-1}, K_{X2} = 17.086 TPa^{-1}, and K_{X3}= 16.315 TPa^{-1} with principal axes $X_1 = b$, $X_2 = c$, and $X_3 = a$. The linear compressibilities (also obtained when the pressure is hydrostatic) of $(F_2BTa)_2PbI_4$ are K_{X1}= 24.4458 TPa^{-1}, K_{X2} = 15.4084 TPa^{-1}, and K_{X3} = 10.6528 TPa^{-1} with principal axes $X_1 = b$, $X_2 = c$, and $X_3 = a$.

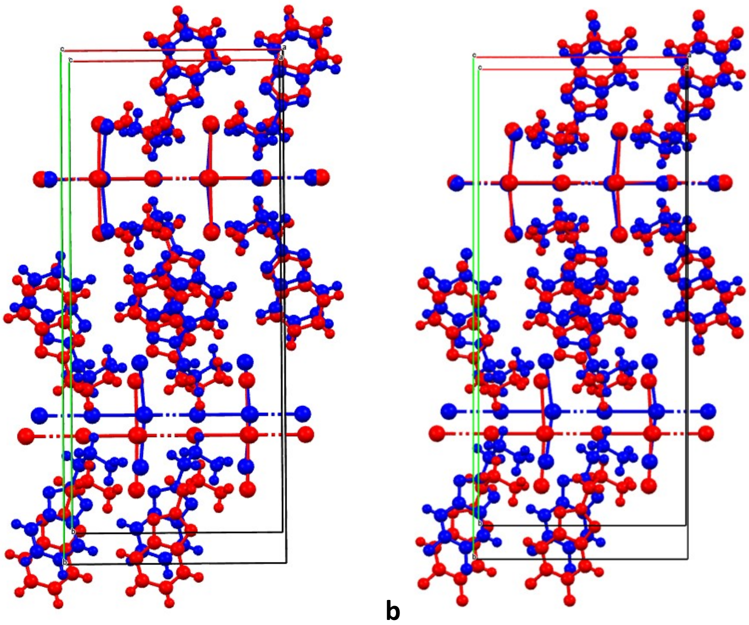

a **b**

Figure S3. (a) Superimposition of the $(BTa)_2PbI_4$ unit cell obtained at ambient pressure (red) and 4.69(4) GPa (blue). (b) Superimposition of the $(F_2BTa)_2PbI_4$ unit cell obtained at ambient pressure (red) and 4.81(2) GPa (blue).

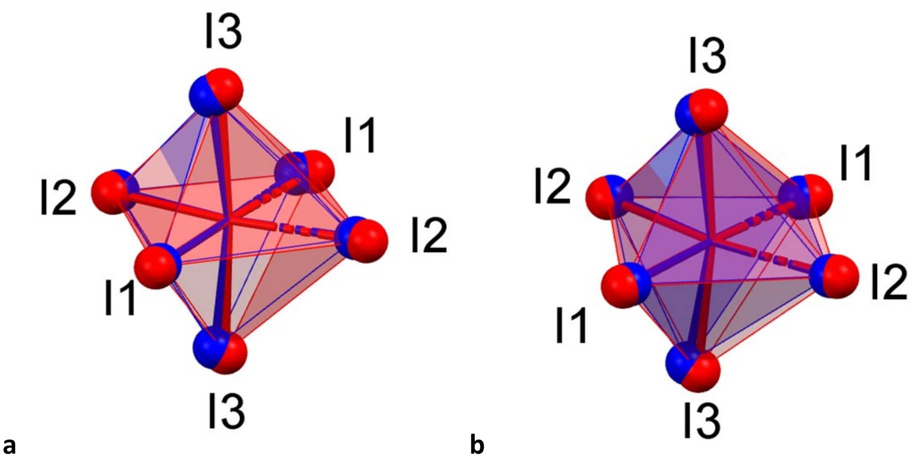

Figure S4. Superimposition of Pb environment in (a) $(BTa)_2PbI_4$ obtained at ambient pressure (red) and 4.69(4) GPa (blue) and in (b) $(F_2BTa)_2PbI_4$ obtained at ambient pressure (red) and 4.81(2) GPa (blue).

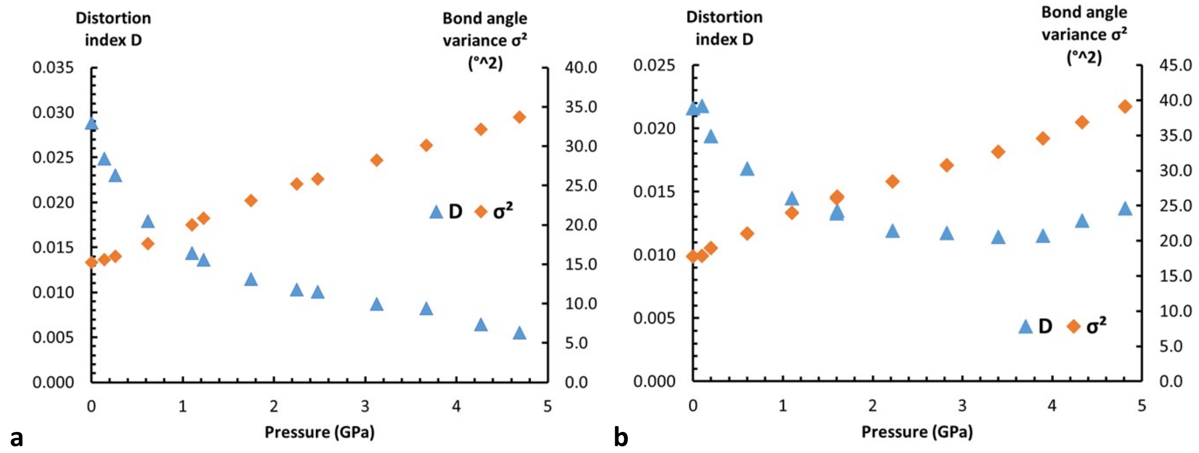

Figure S5. Evolution of octahedron distortion parameters with pressure for (a) $(BTa)_2PbI_4$ and (b) $(F_2BTa)_2PbI_4$. *D* is the Baur length distortion parameter: $D = \frac{1}{6}\sum_{i=1}^{6}\frac{|d_i - d|}{d}$, where d is the average distance in the octahedron. σ² is the bond angle variance: $\sigma^2 = \frac{1}{11}\sum_{i=1}^{12}(\Phi_i - 90)^2$.

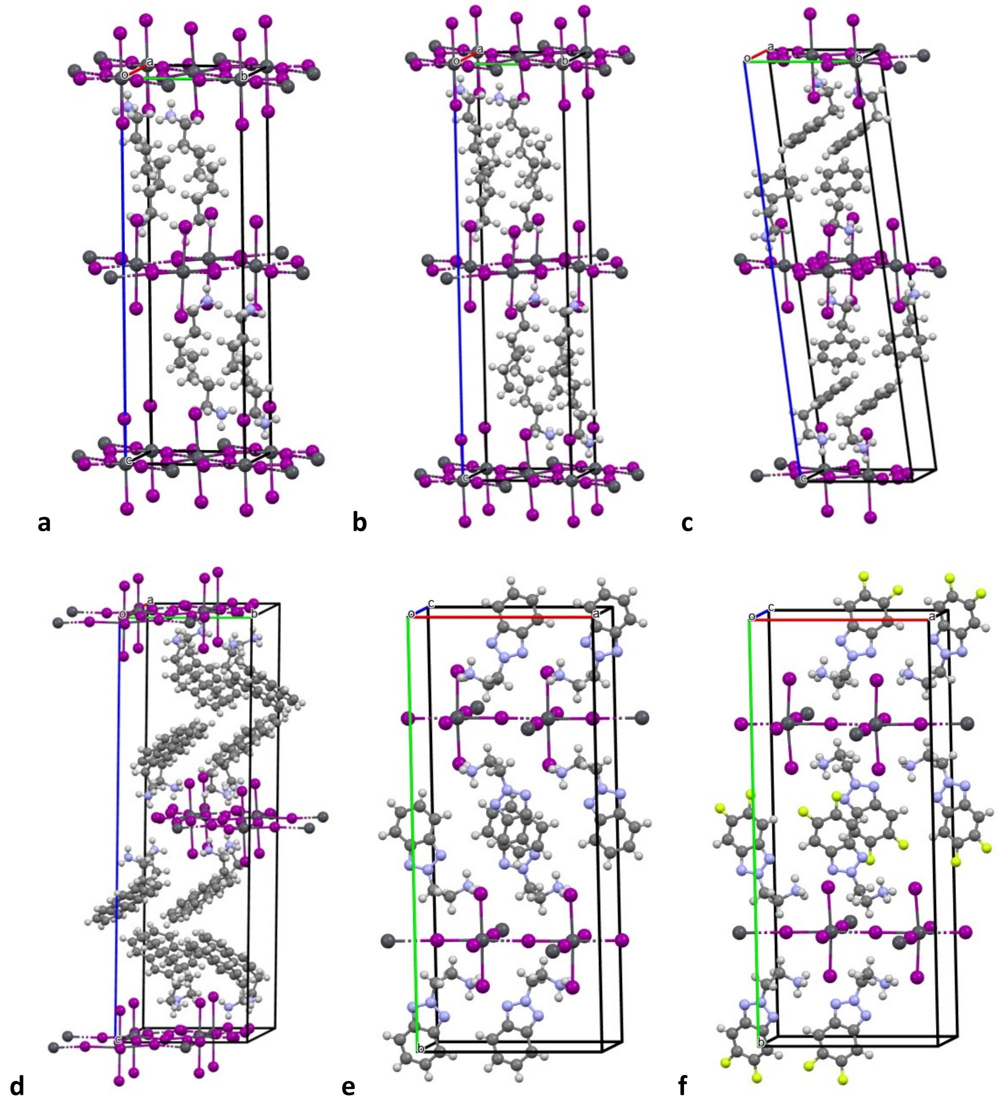

Figure S6. Unit cells of (a) $(BA)_2PbI_4$, (b) $(HA)_2PbI_4$, (c) $(PEA)_2PbI_4$, (d) $(NEA)_2PbI_4$, (e) $(BTa)_2PbI_4$, and (f) $(F_2BTa)_2PbI_4$ at ambient temperature and pressure.

In the cif files, U_{eq} values are provided for each atom in the unit cell. Using this information, an average U_{eq} value can be calculated for each element and for the organic and inorganic sublattice (**Table S4** and **Table S5**).

Table S4. U_{eq} parameters at ambient temperature. The crystal structure of $(PEA)_2PbI_4$ at ambient temperature has been reported by Mitzi's[22] and by Kagan's[23] research group. We chose to use Mitzi's U_{eq} data to compare $(PEA)_2PbI_4$ with the other perovskites.

	U_{eq} (10^{-3} Å^2)						
	**$(BA)_2PbI_4$ (293 K)**	**$(HA)_2PbI_4$ (293 K)**	**$(PEA)_2PbI_4$ (296 K)**	**$(PEA)_2PbI_4$ (300 K)**	**$(NEA)_2PbI_4$ (296 K)**	**$(BTa)_2PbI_4$ (293 K)**	**$(F_2BTa)_2PbI_4$ (293 K)**
	Billing & Lemmerer	Billing & Lemmerer	Mitzi	Kagan	Mitzi	This work	This work
**Pb**	48	44.3	28.68	28.6	33.04	27.48	29.9
**I**	74.7	66.3	41.7	42.8	42	38.9	39.3
**N (NH_3^+)**	94	86	56	59.5	53	38.8	48.7
**C**	205	158	79	66.4	72	34.8	32
**N (core)**						31.1	30.1
**F**							45.3
**Organic**	182.6	147.6	76.3	65.7	70.8	34.2	34.7
**Inorganic**	69.3	61.9	39.1	40	40.3	36.6	37.4

Table S5. U_{eq} parameters of $(PEA)_2PbI_4$,[23] $(BTa)_2PbI_4$, and $(F_2BTa)_2PbI_4$ at 100 K.

	U_{eq} (10^{-3} Å^2)		
	**$(PEA)_2PbI_4$ (100 K)**	**$(BTa)_2PbI_4$ (100 K)**	**$(F_2BTa)_2PbI_4$ (100 K)**
	Kagan	This work	This work
**Pb**	8.05	15.96	10.96
**I**	11.36	20.03	14.14
**C (all)**	24.2	20.1	12.6
**C (tail)**	22.3	21.3	14.9
**C (core)**	24.8	19.7	11.9
**N (NH_3^+)**	17.6	21.3	16.6
**N (core)**		18.2	11.5
**F**			16.8
**Organic**	23.5	19.7	13.3
**Inorganic**	10.7	19	13.3

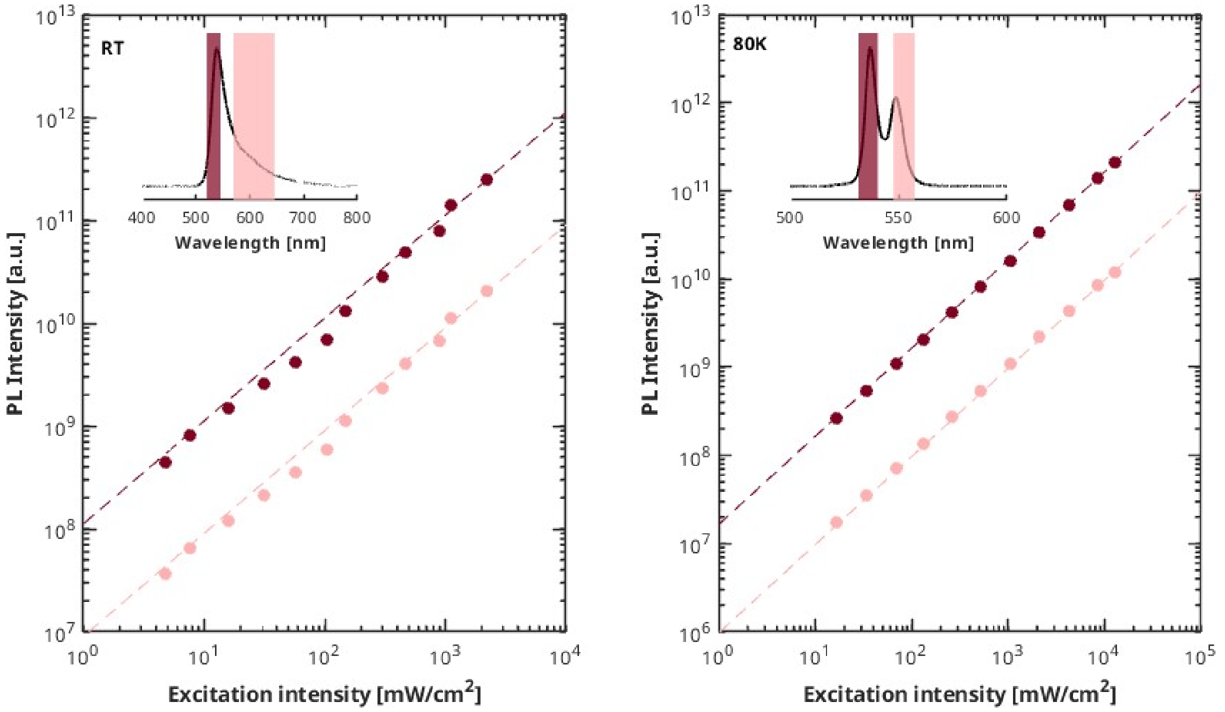

Figure S7. Saturation behaviour of the single-crystal emission of $(FBTa)_2PbI_4$ at room-temperature and 80K. The laser-fluence dependence of surface (purple) and bulk contribution (pink) were obtained by integrating the individual PL spectra over the integration windows indicated in the inset. A linear relation between PL intensity and excitation intensity is characteristic for excitonic emission.

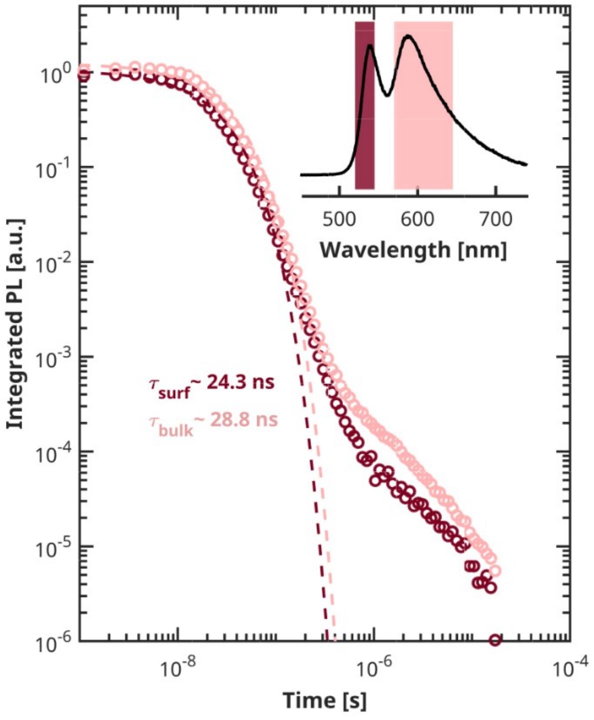

Figure S8. Room-temperature PL decay dynamics of a $(BTa)_2PbI_4$ single crystal. The time-traces were obtained by integrating the individual PL spectra from 525-540 nm (surface contribution) and 570-645 nm (bulk contribution) as indicated in the inset. The dashed lines are single-exponential fits to the PL time-traces with a time constant of 24 ns and 29 ns for the surface and bulk emission, respectively.

We note that the single-crystal PL spectrum shown in **Figure S8** exhibits a more resolved dual-band structure than the PL spectra shown in **Figure 3a** and **Figure 4a** in the main text. This is related to the orientation of the crystal within the cryostat. As reported also in earlier works, the relative intensity between the bulk and surface contributions to the emission spectrum is dependent on the angle between the excitation beam and the crystalline surfaces.[25, 26] Small changes in the sample positioning can hence result in relatively large variations of the overall appearance of the dual-band emission. This does not, however, impact the comparison of the PL decay kinetics obtained for the surface and bulk contributions as presented in **Figure S8**.

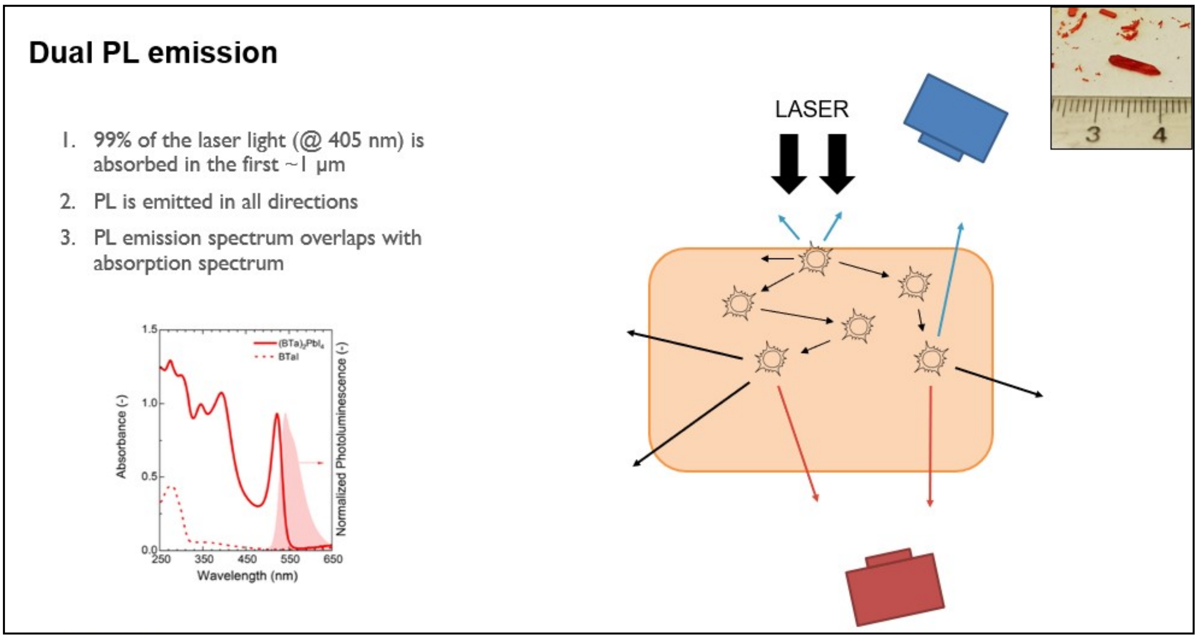

Figure S9. Proposed rationale behind dual PL emission in layered hybrid perovskite single crystals. Thin films of $(BTa)_2PbI_4$, with a thickness of about 0.5 µm, have an absorbance of 1 at 405 nm (the laser excitation wavelength). Through the Lambert-Beer law, this means that a layer of 1 µm, or a single crystal with a thickness of 1 µm, should reach an absorbance of 2 at 405 nm, which means 99% light absorption at 405 nm. Since the single crystals investigated in this work have a thickness of about 1 mm, this indicates that **all the laser light is absorbed in the first 0.1 % of the crystal thickness**. In other words, the surface layers of the $(BTa)_2PbI_4$ crystal absorb all the laser light. Additionally, **PL emission is emitted in equal amounts in all directions**, i.e. in the direction out of the crystal as well as in the direction deeper into the crystal bulk. Finally, we observe that the **absorption and PL emission spectrum overlap**, indicating that PL emission towards the interior of the crystal can be reabsorbed, especially given the theoretical calculations in **Figure 3b**, which reveal a smaller band gap for the crystal bulk than for the crystal surface. Instead of photon reabsorption ("photon recycling"), it is also possible that the surface decay and bulk excitation transition dipoles couple in the form of Förster resonance energy transfer (FRET), yielding a faster energy transfer from the surface layers to the bulk layers.

Hence, if the detector and the laser are placed at the same side of the crystal, both surface and bulk PL emission are observed, whereas if the detector and the laser are placed at opposite sides of the crystal, only the bulk PL emission is observed.

Table S6. Best-fit parameters for the Gaussian (σ) and Lorentzian (γ) line broadening of the Voigtian fit to the high-energy flank of the temperature-dependent PL of $(BTa)_2PbI_4$, $(F_2BTa)_2PbI_4$ and $(PEA)_2PbI_4$ shown in **Figure 4b** in the main text.

	**$(BTa)_2PbI_4$**		**$(F_2BTa)_2PbI_4$**		**$(PEA)_2PbI_4$**	
***T* [K]**	***σ* [meV]**	***γ* [meV]**	***σ* [meV]**	***γ* [meV]**	***σ* [meV]**	***γ* [meV]**
**80**	8.7	4.0	6.8	5.3	3.6	6.2
**100**	12.5	5.7	9.1	7.6	3.9	9.7
**120**	13.8	6.4	10.6	8.5	4.1	11.2
**140**	16.1	6.8	12.5	9.1	4.3	12.2
**160**	18.6	7.4	14.7	9.3	4.7	13.5
**180**	22.0	7.1	17.6	9.2	4.9	14.8
**200**	25.2	6.9	20.8	8.8	6.0	16.1
**220**	28.2	6.6	24.0	8.0	9.0	17.6
**240**	32.0	5.5	27.1	7.2	10.3	18.1
**260**	33.0	7.2	30.0	7.0	12.0	18.9
**280**	34.4	8.4	34.1	6.8	13.8	19.4
**RT**	38.3	7.2	37.6	6.7	14.9	19.6

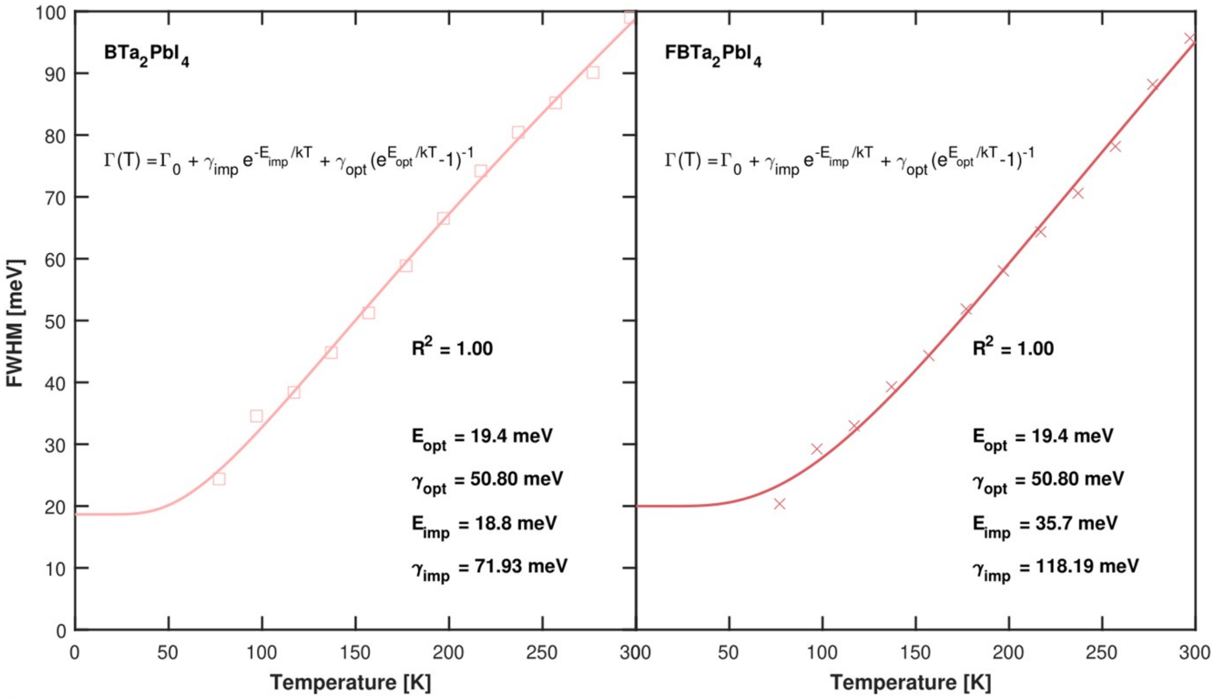

Figure S10. Temperature dependence of the emission line widths extracted from the single-crystal PL data in Figure 4 of the main text. The solid lines are best fits of the thermal line broadening with eq (1), wherein the parameters for the optical phonon scattering were kept fixed.

Despite the Gaussian line shape of the PL spectra of the benzotriazole perovskite single crystals at ambient temperature, it cannot be fully excluded that scattering from optical phonons still presents a significant contribution to the total line broadening. To demonstrate that this would, however, not qualitatively impact our analysis in the main text, we have performed an additional fit of the temperature-dependent line broadening of the PL emission where both impurity scattering and optical phonon scattering are considered. **Figure S10** shows the best fit of the thermal line broadening for $(BTa)_2PbI_4$ and $(F_2BTa)_2PbI_4$ with the following equation:

$$\Gamma(T) = \Gamma_0 + \frac{\gamma_{\mathrm{opt}}}{e^{E_{\mathrm{opt}}/kT} - 1} + \gamma_{\mathrm{imp}} e^{E_{\mathrm{imp}}/kT} \quad (1)$$

where γ_{opt} and γ_{imp} are the coupling terms describing scattering from optical phonons and ionizable impurities, respectively. E_{opt} is the mean energy of the optical phonons, E_{imp} the ionization energy of the impurity donor or acceptor. To avoid overfitting of the data, the parameters for the exciton-phonon interaction were kept fixed at the best-fit result previously obtained for $(PEA)_2PbI_4$, i.e. γ_{opt} = 50.8 meV and E_{opt} = 19.4 meV. The resulting best-fit parameters for the impurity scattering are shown in **Figure S10**. Since the coupling constant directly scales with the contribution of impurity scattering to the total line broadening, the obtained coupling constants are about half of those when no contribution due to optical phonon scattering to the total line width is assumed. Yet, more importantly, the obtained ionization energies for the impurities are in very good agreement with those presented in the main text: E_{imp} = 19 meV (with phonon scattering) versus E_{imp} = 27 meV (without phonon scattering) for $(BTa)_2PbI_4$ and E_{imp} = 36 meV (with phonon scattering) versus E_{imp} = 34 meV (without phonon scattering) for $(F_2BTa)_2PbI_4$. Hence the conclusion that interactions with very shallow defects are a dominant PL line broadening mechanism in the benzotriazole perovskite single crystals still holds. Note that we cannot determine from our data if the exciton-phonon interaction parameters of the benzotriazole perovskites are similar to those of $(PEA)_2PbI_4$; these values were simply chosen as a reasonable starting point for the current analysis. Indeed, as shown in the main text, the data can be fitted very well when only impurity scattering is considered.

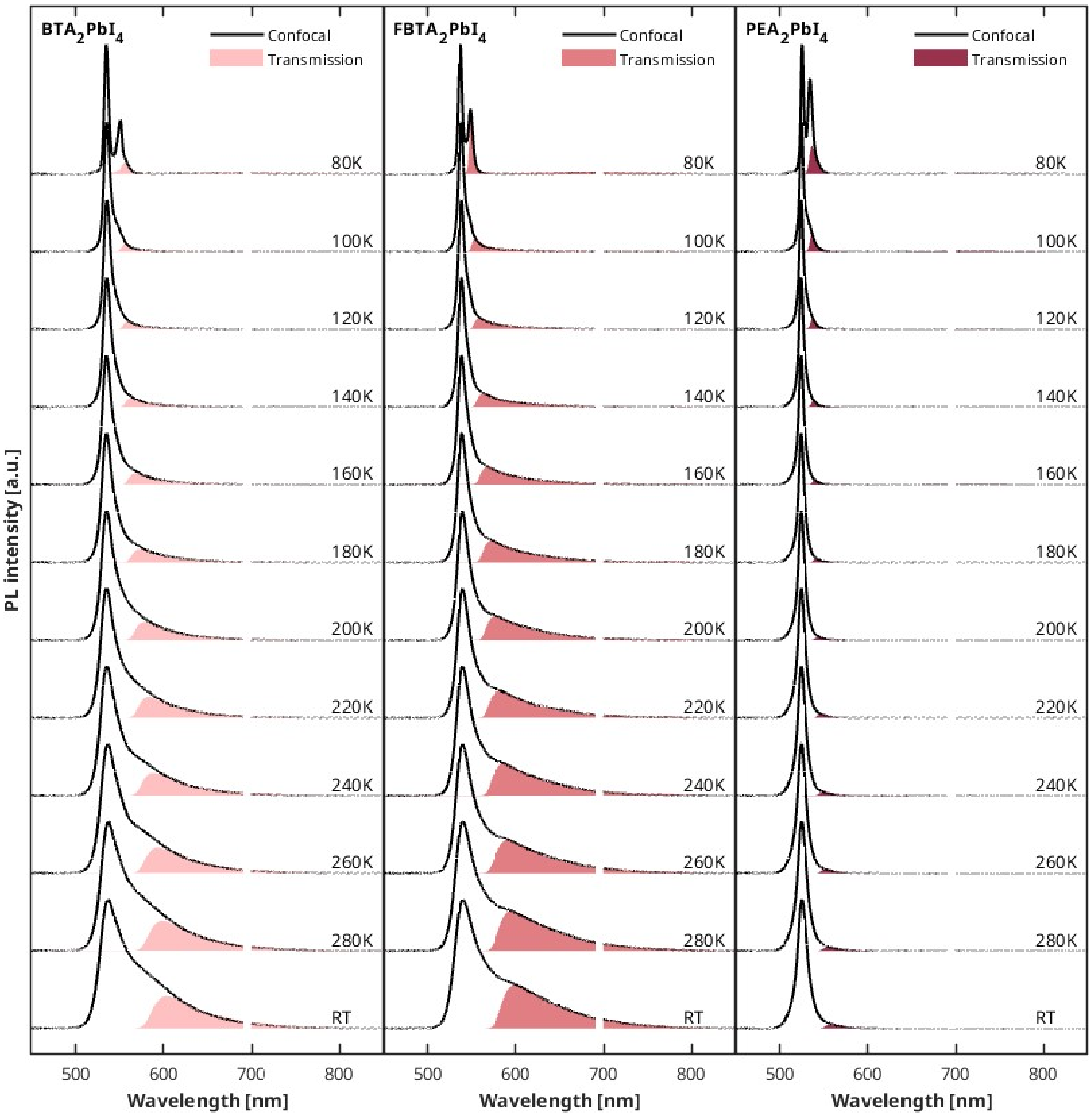

Figure S11. Temperature-dependent PL of the $(BTa)_2PbI_4$, $(F_2BTa)_2PbI_4$ and $(PEA)_2PbI_4$ single crystals under excitation with 3.06 eV (405 nm). Comparison of the PL collected by two different measurement geometries: confocal (solid line) and transmission geometry (shaded area), see main text for details. Around 690 nm, a narrow artifact due to a luminescent impurity in the cryostat window has been removed from all spectra.

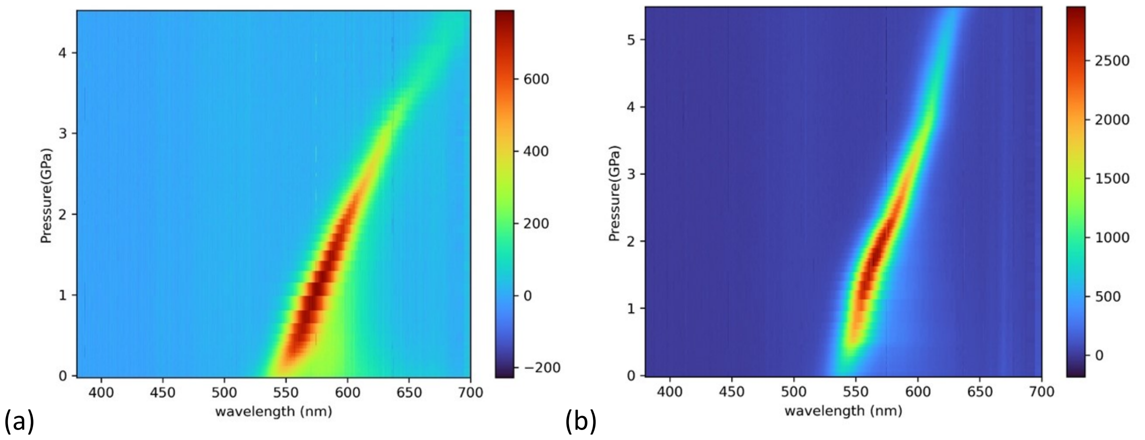

Figure S12. PL emission shift of (a) $(BTa)_2PbI_4$ and (b) $(F_2BTa)_2PbI_4$ with increasing pressure.

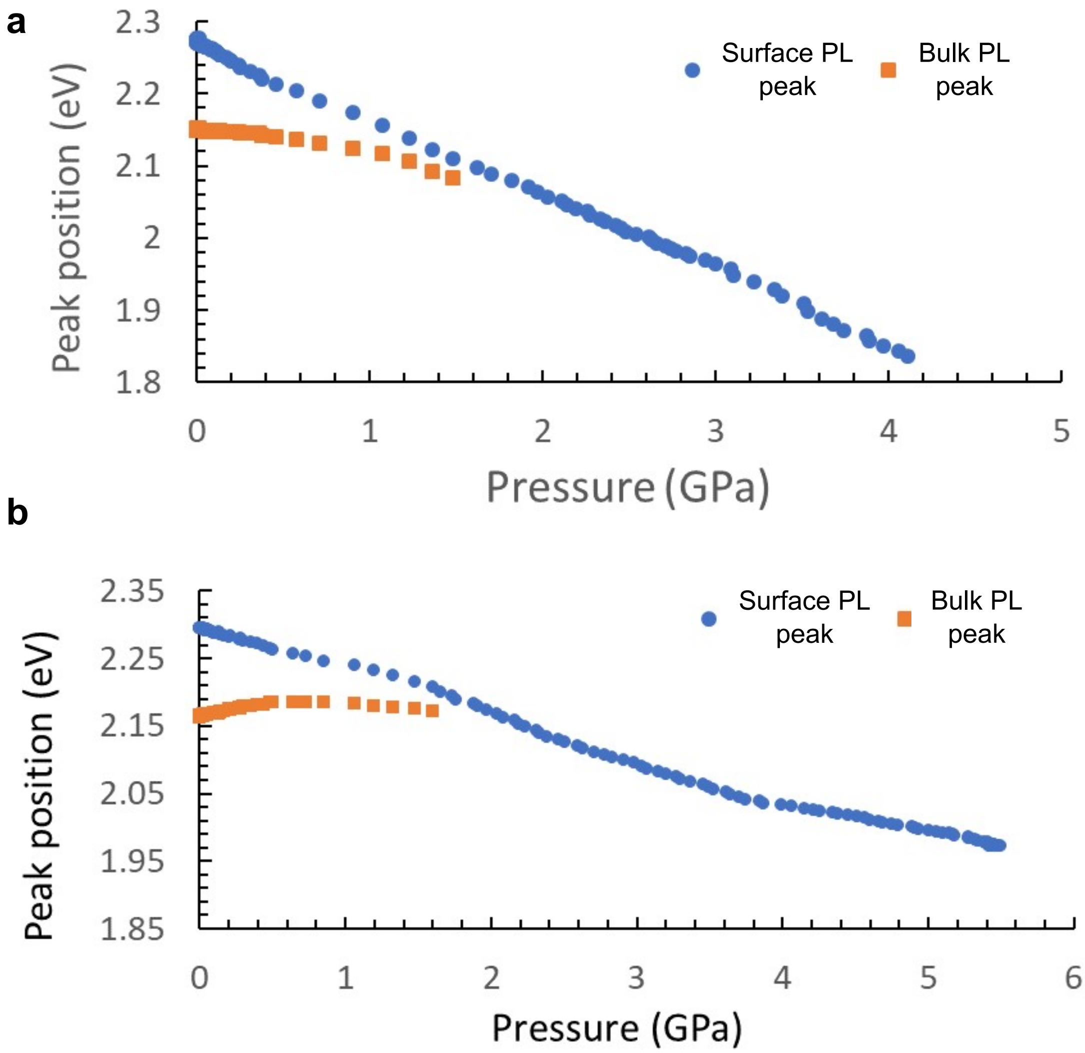

Figure S13. Evolution of the PL emission maxima with increasing pressure for (a) $(BTa)_2PbI_4$ and (b) $(F_2BTa)_2PbI_4$.

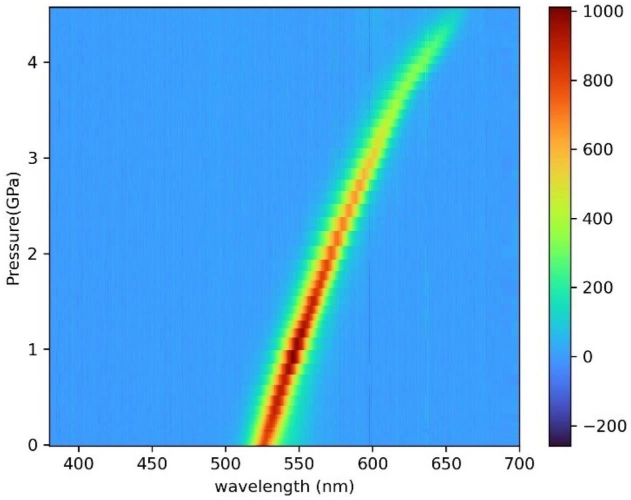

Figure S14. PL emission shift of $(PEA)_2PbI_4$ with increasing pressure.

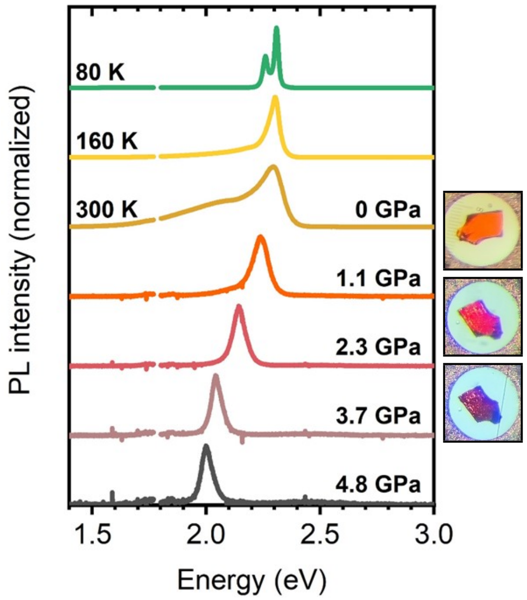

Figure S15. PL emission shift of a $(F_2BTa)_2PbI_4$ single crystal with temperature and with pressure, causing the crystal to change color.

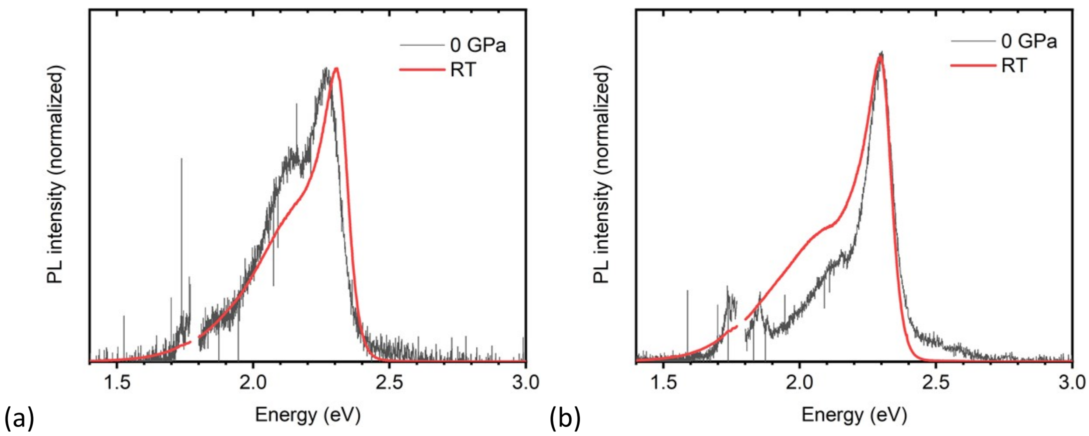

Figure S16. Comparison of the PL spectra at ambient conditions in the temperature experiment (RT) and in the pressure experiment (0 GPa) on a single crystal of (a) $(BTa)_2PbI_4$ and (b) $(F_2BTa)_2PbI_4$.

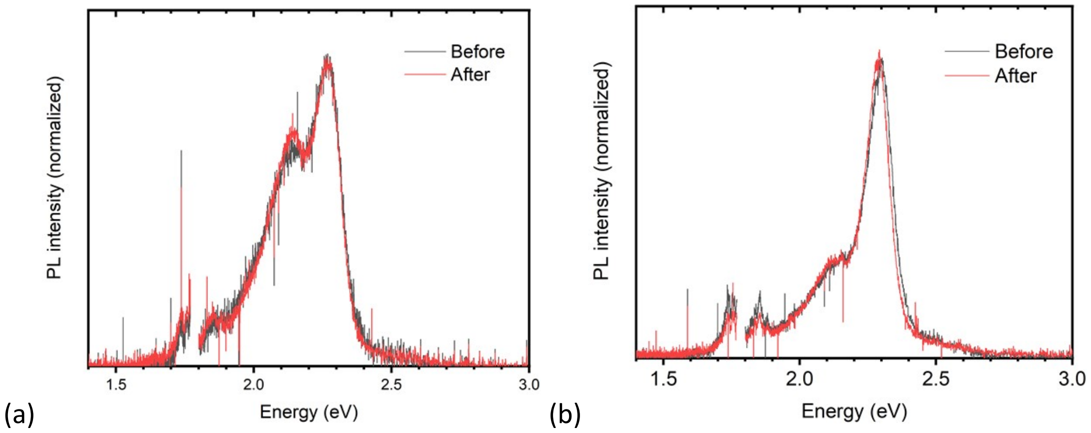

Figure S17. Comparison of the PL spectrum before and after a pressure sweep from 0 GPa to 5 GPa and back to 0 GPa on a single crystal of (a) $(BTa)_2PbI_4$ and (b) $(F_2BTa)_2PbI_4$.

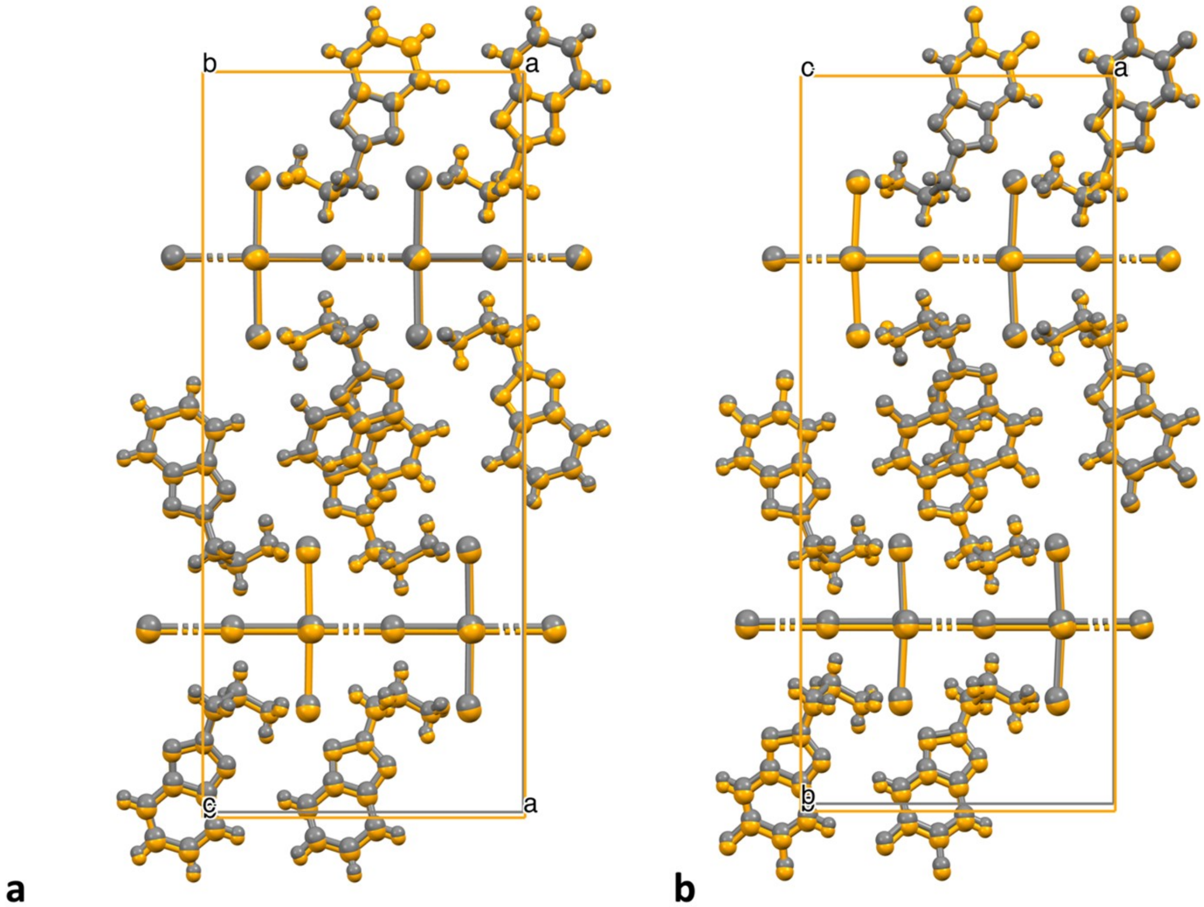

Figure S18. (a) Superimposition of the unit cell of $(BTa)_2PbI_4$ obtained at 100 K (grey) and 293 K (orange). (b) Superimposition of the unit cell of $(F_2BTa)_2PbI_4$ obtained at 100 K (grey) and 293 K (orange).

Table S7. Data collection and structure refinement parameters for the structures of $(BTa)_2PbI_4$ (100 K), $(BTa)_2PbI_4$ (293 K), $(F_2BTa)_2PbI_4$ (100 K) and $(F_2BTa)_2PbI_4$ (293 K).

	$(BTa)_2PbI_4$	$(BTa)_2PbI_4$	$(F_2BTa)_2PbI_4$	$(F_2BTa)_2PbI_4$
Molecular formula	$C_{16}H_{22}I_4N_8Pb$	$C_{16}H_{22}I_4N_8Pb$	$C_{16}H_{18}F_4I_4N_8Pb$	$C_{16}H_{18}F_4I_4N_8Pb$
Formula weight ($gmol^{-1}$)	1041.2	1041.2	1113.2	1113.2
T (K)	100	293	100	293
Crystal system	orthorhombic	orthorhombic	orthorhombic	orthorhombic
Space group	*P*nma (No. 62)	*P*nma (No. 62)	*P*nma (No. 62)	*P*nma (No. 62)
a (Å)	12.8383(2)	12.9102(8)	12.8517(2)	12.9256(6)
b (Å)	29.6464(4)	29.8736(15)	29.9017(5)	30.2098(14)
c (Å)	6.54000(10)	6.6182(4)	6.57340(10)	6.6341(3)
V ($Å^3$)	2489.19(6)	2552.5(3)	2526.08(7)	2590.5(2)
Z	4	4	4	4
ρ_{calc} (gcm^{-3})	2.778	2.709	2.927	2.854
$2\theta_{max}$ (°)	59.322	57.530	59.316	56.988
F(000)	1872	1872	2000	2000
Measured reflections	38909	11295	26997	14054
Unique reflections	3422	2966	3350	2913
Observed reflections ($I > 2\sigma(I)$)	3156	2105	3025	1902
Parameters refined	137	138	155	155
R_1	0.0254	0.0368	0.0210	0.0387
wR_2	0.0593	0.0645	0.0412	0.0513
R_1 (all data)	0.0291	0.0649	0.0261	0.0823
wR_2 (all data)	0.0614	0.0761	0.0432	0.0612
Goodness-of-fit (GOF)	1.053	1.043	1.076	0.995
μ (mm^{-1})	11.759	11.467	11.619	11.330

Table S8. Selected equivalent bond lengths (Å) and bond angles (°) for the structures of $(BTa)_2PbI_4$ (100 K), $(BTa)_2PbI_4$ (293 K), $(F_2BTa)_2PbI_4$ (100 K) and $(F_2BTa)_2PbI_4$ (293 K).

	$(BTa)_2PbI_4$	$(BTa)_2PbI_4$	$(F_2BTa)_2PbI_4$	$(F_2BTa)_2PbI_4$
T (K)	100	293	100	293
Pb(1)-I(1) (Å)	3.0749(4)	3.1086(8)	3.1341(4)	3.1876(8)
Pb(1)-I(2) (Å)	3.1654(6)	3.1748(6)	3.1521(6)	3.1564(6)
Pb(1)-I(3) (Å)	3.1947(4)	3.2337(8)	3.2052(3)	3.2583(8)
Pb(1)-I(1)$^{i, ii}$ (Å)	3.3829(4)	3.3796(8)	3.3279(4)	3.3058(8)
Pb(1)-I(2)^{iii} (Å)	3.1654(6)	3.1748(6)	3.1521(6)	3.1564(6)
Pb(1)-I(3)$^{iv, v}$ (Å)	3.3492(4)	3.3879(8)	3.3736(3)	3.3790(8)
Pb(1)-I(1)-Pb(1)$^{i, ii}$ (°)	173.38(1)	173.76(3)	177.91(1)	178.61(3)
Pb(1)-I(3)-Pb(1)$^{iv, v}$ (°)	176.08(1)	176.33(3)	175.34(1)	176.46(2)

Symmetry codes: i = -1/2+x, 3/2-y,1/2-z; ii = 1/2+x, 3/2-y, 1/2-z; iii = x, 3/2-y, z; iv = x, y, 1+z; v = x, y, -1+z.

7. References

1. Maufort, A.; Cerdá, J.; Van Hecke, K.; Deduytsche, D.; Verding, A.; Ruttens, B.; Li, W.; Detavernier, C.; Lutsen, L.; Quarti, C.; Vanderzande, D.; Beljonne, D.; Van Gompel, W. T. M., Elucidating the Non-Covalent Interactions that Trigger Interdigitation in Lead-Halide Layered Hybrid Perovskites. *Inorganic Chemistry* **2024,** *63* (12), 5568-5579.
2. Caiazzo, A.; Maufort, A.; van Gorkom, B. T.; Remmerswaal, W. H. M.; Orri, J. F.; Li, J.; Wang, J.; van Gompel, W. T. M.; Van Hecke, K.; Kusch, G.; Oliver, R. A.; Ducati, C.; Lutsen, L.; Wienk, M. M.; Stranks, S. D.; Vanderzande, D.; Janssen, R. A. J., 3D Perovskite Passivation with a Benzotriazole-Based 2D Interlayer for High-Efficiency Solar Cells. *ACS Applied Energy Materials* **2023,** *6* (7), 3933-3943.
3. Fateev, S. A.; Petrov, A. A.; Ordinartsev, A. A.; Grishko, A. Y.; Goodilin, E. A.; Tarasov, A. B., Universal Strategy of 3D and 2D Hybrid Perovskites Single Crystal Growth via In Situ Solvent Conversion. *Chemistry of Materials* **2020,** *32* (22), 9805-9812.
4. Dolomanov, O. V.; Bourhis, L. J.; Gildea, R. J.; Howard, J. A. K.; Puschmann, H., OLEX2: a complete structure solution, refinement and analysis program. *Journal of Applied Crystallography* **2009,** *42* (2), 339-341.
5. Sheldrick, G., SHELXT - Integrated space-group and crystal-structure determination. *Acta Crystallographica Section A* **2015,** *71* (1), 3-8.
6. Sheldrick, G., Crystal structure refinement with SHELXL. *Acta Crystallographica Section C* **2015,** *71* (1), 3-8.
7. Piermarini, G. J.; Block, S., Ultrahigh pressure diamond-anvil cell and several semiconductor phase transition pressures in relation to the fixed point pressure scale. *Review of Scientific Instruments* **1975,** *46* (8), 973-979.
8. Murata, K.; Yokogawa, K.; Yoshino, H.; Klotz, S.; Munsch, P.; Irizawa, A.; Nishiyama, M.; Iizuka, K.; Nanba, T.; Okada, T.; Shiraga, Y.; Aoyama, S., Pressure transmitting medium Daphne 7474 solidifying at 3.7 GPa at room temperature. *Rev Sci Instrum* **2008,** *79* (8), 085101.

9. Vijayakumar-Syamala, V.; Aubert, E.; Deutsch, M.; Wenger, E.; Dhaka, A.; Fourmigue, M.; Nespolo, M.; Espinosa, E., N-Iodosaccharin-pyridine co-crystal system under pressure: experimental evidence of reversible twinning. *Acta Crystallographica Section B* **2022,** *78* (3 Part 2), 436-449.
10. Dawson, A.; Allan, D. R.; Parsons, S.; Ruf, M., Use of a CCD diffractometer in crystal structure determinations at high pressure. *Journal of Applied Crystallography* **2004,** *37* (3), 410-416.
11. Krause, L.; Herbst-Irmer, R.; Sheldrick, G. M.; Stalke, D., Comparison of silver and molybdenum microfocus X-ray sources for single-crystal structure determination. *Journal of Applied Crystallography* **2015,** *48* (1), 3-10.
12. Angel, R. J., Equations of State. In *High-Temperature and High Pressure Crystal Chemistry*, Hazen, R. M.; Downs, R. T., Eds. De Gruyter: **2000**; pp 35-60.
13. Gonzalez-Platas, J.; Alvaro, M.; Nestola, F.; Angel, R., EosFit7-GUI: a new graphical user interface for equation of state calculations, analyses and teaching. *Journal of Applied Crystallography* **2016,** *49* (4), 1377-1382.
14. Giannozzi, P.; Andreussi, O.; Brumme, T.; Bunau, O.; Buongiorno Nardelli, M.; Calandra, M.; Car, R.; Cavazzoni, C.; Ceresoli, D.; Cococcioni, M.; Colonna, N.; Carnimeo, I.; Dal Corso, A.; de Gironcoli, S.; Delugas, P.; DiStasio, R. A.; Ferretti, A.; Floris, A.; Fratesi, G.; Fugallo, G.; Gebauer, R.; Gerstmann, U.; Giustino, F.; Gorni, T.; Jia, J.; Kawamura, M.; Ko, H. Y.; Kokalj, A.; Küçükbenli, E.; Lazzeri, M.; Marsili, M.; Marzari, N.; Mauri, F.; Nguyen, N. L.; Nguyen, H. V.; Otero-de-la-Roza, A.; Paulatto, L.; Poncé, S.; Rocca, D.; Sabatini, R.; Santra, B.; Schlipf, M.; Seitsonen, A. P.; Smogunov, A.; Timrov, I.; Thonhauser, T.; Umari, P.; Vast, N.; Wu, X.; Baroni, S., Advanced capabilities for materials modelling with Quantum ESPRESSO. *Journal of Physics: Condensed Matter* **2017,** *29* (46), 465901.
15. Giannozzi, P.; Baroni, S.; Bonini, N.; Calandra, M.; Car, R.; Cavazzoni, C.; Ceresoli, D.; Chiarotti, G. L.; Cococcioni, M.; Dabo, I.; Dal Corso, A.; de Gironcoli, S.; Fabris, S.; Fratesi, G.; Gebauer, R.; Gerstmann, U.; Gougoussis, C.; Kokalj, A.; Lazzeri, M.; Martin-Samos, L.; Marzari, N.; Mauri, F.; Mazzarello, R.; Paolini, S.; Pasquarello, A.; Paulatto, L.; Sbraccia, C.; Scandolo, S.; Sclauzero, G.; Seitsonen, A. P.; Smogunov, A.; Umari, P.; Wentzcovitch, R. M., QUANTUM ESPRESSO: a modular and open-source software project for quantum simulations of materials. *Journal of Physics: Condensed Matter* **2009,** *21* (39), 395502.
16. Giannozzi, P.; Baseggio, O.; Bonfà, P.; Brunato, D.; Car, R.; Carnimeo, I.; Cavazzoni, C.; de Gironcoli, S.; Delugas, P.; Ferrari Ruffino, F.; Ferretti, A.; Marzari, N.; Timrov, I.; Urru, A.; Baroni, S., Quantum ESPRESSO toward the exascale. *The Journal of Chemical Physics* **2020,** *152* (15), 154105.
17. Perdew, J. P.; Burke, K.; Ernzerhof, M., Generalized Gradient Approximation Made Simple. *Physical Review Letters* **1996,** *77* (18), 3865-3868.
18. van Setten, M. J.; Giantomassi, M.; Bousquet, E.; Verstraete, M. J.; Hamann, D. R.; Gonze, X.; Rignanese, G. M., The PseudoDojo: Training and grading a 85 element optimized norm-conserving pseudopotential table. *Computer Physics Communications* **2018,** *226*, 39-54.
19. Monkhorst, H. J.; Pack, J. D., Special points for Brillouin-zone integrations. *Physical Review B* **1976,** *13* (12), 5188-5192.
20. Grimme, S.; Antony, J.; Ehrlich, S.; Krieg, H., A consistent and accurate ab initio parametrization of density functional dispersion correction (DFT-D) for the 94 elements H-Pu. *The Journal of Chemical Physics* **2010,** *132* (15), 154104.
21. Billing, D. G.; Lemmerer, A., Synthesis, characterization and phase transitions in the inorganic-organic layered perovskite-type hybrids [(CnH2n+1NH3)2PbI4], n = 4, 5 and 6. *Acta Crystallogr B* **2007,** *63* (Pt 5), 735-47.
22. Du, K.-z.; Tu, Q.; Zhang, X.; Han, Q.; Liu, J.; Zauscher, S.; Mitzi, D. B., Two-Dimensional Lead(II) Halide-Based Hybrid Perovskites Templated by Acene Alkylamines: Crystal Structures, Optical Properties, and Piezoelectricity. *Inorganic Chemistry* **2017,** *56* (15), 9291-9302.
23. Straus, D. B.; Iotov, N.; Gau, M. R.; Zhao, Q.; Carroll, P. J.; Kagan, C. R., Longer Cations Increase Energetic Disorder in Excitonic 2D Hybrid Perovskites. *The Journal of Physical Chemistry Letters* **2019,** *10* (6), 1198-1205.

24. Ma, D.; Fu, Y.; Dang, L.; Zhai, J.; Guzei, I. A.; Jin, S., Single-crystal microplates of two-dimensional organic–inorganic lead halide layered perovskites for optoelectronics. *Nano Research* **2017,** *10* (6), 2117-2129.
25. Du, Q.; Zhu, C.; Yin, Z.; Na, G.; Cheng, C.; Han, Y.; Liu, N.; Niu, X.; Zhou, H.; Chen, H.; Zhang, L.; Jin, S.; Chen, Q., Stacking Effects on Electron–Phonon Coupling in Layered Hybrid Perovskites via Microstrain Manipulation. *ACS Nano* **2020,** *14* (5), 5806-5817.
26. Sheikh, T.; Shinde, A.; Mahamuni, S.; Nag, A., Possible Dual Bandgap in (C4H9NH3)2PbI4 2D Layered Perovskite: Single-Crystal and Exfoliated Few-Layer. *ACS Energy Letters* **2018,** *3* (12), 2940-2946.
